# Using joint probability density to create most informative unidimensional indices: a new method using pain and psychiatric severity as examples

**DOI:** 10.1186/s12874-024-02299-y

**Published:** 2024-08-06

**Authors:** Siamak Noorbaloochi, Barbara A. Clothier, Maureen Murdoch

**Affiliations:** 1 Center for Care Delivery and Outcomes Research, Minneapolis VA Health Care System, One Veterans Drive (152), Minneapolis, MN 55417 USA; 2 Department of Internal Medicine, University of Minnesota Medical School, 420 Delaware St SE, Minneapolis, MN 55455 USA; 3 Section of General Internal Medicine, Minneapolis VA Health Care System, One Veterans Drive (111-0), Minneapolis, MN 55417 USA

**Keywords:** Joint probability density, Psychometrics, IRT models, Pain, Psychiatric illness, Composite score, Bayesian network, Brief pain inventory, Manifestations of psychiatric illness severity index

## Abstract

**Background:**

Dimension reduction methods do not always reduce their underlying indicators to a single composite score. Furthermore, such methods are usually based on optimality criteria that require discarding some information. We suggest, under some conditions, to use the joint probability density function (joint pdf or JPD) of p-dimensional random variable (the p indicators), as an index or a composite score. It is proved that this index is more informative than any alternative composite score. In two examples, we compare the JPD index with some alternatives constructed from traditional methods.

**Methods:**

We develop a probabilistic unsupervised dimension reduction method based on the probability density of multivariate data. We show that the conditional distribution of the variables given JPD is uniform, implying that the JPD is the most informative scalar summary under the most common notions of information. B. We show under some widely plausible conditions, JPD can be used as an index. To use JPD as an index, in addition to having a plausible interpretation, all the random variables should have approximately the same direction(unidirectionality) as the density values (codirectionality). We applied these ideas to two data sets: first, on the 7 Brief Pain Inventory Interference scale (BPI-I) items obtained from 8,889 US Veterans with chronic pain and, second, on a novel measure based on administrative data for 912 US Veterans. To estimate the JPD in both examples, among the available JPD estimation methods, we used its conditional specifications, identified a well-fitted parametric model for each factored conditional (regression) specification, and, by maximizing the corresponding likelihoods, estimated their parameters. Due to the non-uniqueness of conditional specification, the average of all estimated conditional specifications was used as the final estimate. Since a prevalent common use of indices is ranking, we used measures of monotone dependence [e.g., Spearman’s rank correlation (rho)] to assess the strength of unidirectionality and co-directionality. Finally, we cross-validate the JPD score against variance–covariance-based scores (factor scores in unidimensional models), and the “person’s parameter” estimates of (Generalized) Partial Credit and Graded Response IRT models. We used Pearson Divergence as a measure of information and Shannon’s entropy to compare uncertainties (informativeness) in these alternative scores.

**Results:**

An unsupervised dimension reduction was developed based on the joint probability density (JPD) of the multi-dimensional data. The JPD, under regularity conditions, may be used as an index. For the well-established Brief Pain Interference Inventory (BPI-I (the short form with 7 Items) and for a new mental health severity index (MoPSI) with 6 indicators, we estimated the JPD scoring. We compared, assuming unidimensionality, factor scores, Person’s scores of the Partial Credit model, the Generalized Partial Credit model, and the Graded Response model with JPD scoring. As expected, all scores’ rankings in both examples were monotonically dependent with various strengths. Shannon entropy was the smallest for JPD scores. Pearson Divergence of the estimated densities of different indices against uniform distribution was maximum for JPD scoring.

**Conclusions:**

An unsupervised probabilistic dimension reduction is possible. When appropriate, the joint probability density function can be used as the most informative index. Model specification and estimation and steps to implement the scoring were demonstrated. As expected, when the required assumption in factor analysis and IRT models are satisfied, JPD scoring agrees with these established scores. However, when these assumptions are violated, JPD scores preserve all the information in the indicators with minimal assumption.

**Supplementary Information:**

The online version contains supplementary material available at 10.1186/s12874-024-02299-y.

## Introduction

In this paper we introduce an *unsupervised probabilistic* method for dimension reduction (DR) particularly when it results in a single composite score or an index.

Sufficient dimension reduction (SDR), as a supervised probabilistic approach, is based on the conditional densities [1–4] to construct the q summaries. It requires:

$$f\left(y|{x}_{1},{x}_{2},\dots ,{x}_{p}\right)=f\left(y|{s}_{1},{s}_{2},\dots ,{s}_{q}\right)$$, where $${{s}_{k}={h}_{k}(x}_{1},{x}_{2},\dots ,{x}_{p}) , k=\text{1,2},..q.$$

To extend this idea to unsupervised situations, we use the *joint density* of predictors and replace the sufficiency of summaries, i.e., being the most informative for the response, with the notion of most informativeness. Thus, formally we define:

### Definition

The function ${\varvec{s}}:{{\varvec{R}}}^{{\varvec{p}}}\to {{\varvec{R}}}^{{\varvec{q}}},\boldsymbol{ }{{s}_{k}={h}_{k}(x}_{1},{x}_{2},\dots ,{x}_{p}) , k=\text{1,2},..q.$ is a most informative summary if:

$f\left({x}_{1},{x}_{2},\dots ,{x}_{p}|{s}_{1},{s}_{2},\dots ,{s}_{q}\right)$ is a uniform distribution, for each

1 $${\varvec{s}}=\left(s_1,s_2,\dots,s_q\right).$$

In other words, given the summary score, the conditional distribution of the indicators should have maximum entropy and hence minimum information. Note that this means ${\varvec{s}}$ contains all the information in the indicators. Note that $f\left({x}_{1},{x}_{2},\dots ,{x}_{p}\right)$, the joint probability density (JPD) function, satisfies (1) (see the appendix for a proof).

The following set of necessary conditions are required for JPD summary to be an index:

First, ${x}_{1}, {x}_{2},\dots ,{x}_{p}$, must be at least ordinal be conceptually related, and undergird a broader concept, such as pain experience, mental health status, volatility of the stock market, and as such. We refer to this property as *relevance*. In factor analysis language, the latent space of ${x}_{1}, {x}_{2},\dots ,{x}_{p}$ indicators should be unidimensional. Second, all indicators must be recorded or coded such that their order (higher or lower) values agree, and their pairwise monotone correlations be strong and in the same direction (sign). We call this property the *unidirectionality* of the indicators. Third, the index, $s,$ should be monotonically correlated with each of the indicators (variables) and with the same sign. We refer to this property as the *codirectionality* of the summary with the variables. Strength of relevance and unidirectionality can be achieved at the study design stage where one decides what indicators should be collected or how they should be measured. The strength of codirectionality depends on actual frequencies and how the density values change by changes in indicators’ value patterns.

Many unsupervised techniques optimize some aspect of variables’ joint probability density or joint probability mass function. For example, Principal Components Analysis [5], its kernelized versions [6, 7] and Factor Analysis are based on the variances and the covariances of the joint distribution, respectively. Item Response Theory (IRT) uses the joint pdf of indicators; however IRT benefits from four assumptions: local independence, correctness of functional form, usually a linear decomposition of the model parameters, the assumption that a unique parameter, called ability that measures subjects differences, and that this latent variable has a normal distribution in the population [though see multi-dimensional IRT [8] and nonparametric IRT [9] for some relaxations]. The local independence assumption permits one to write the joint pdf as a product of the univariates indicators’ (items’) pdfs given the parameter values.

Using the joint probability density, or a monotone transformation of it, as an index offers many advantages. Variables(indicators) do not need to meet restrictive assumptions beyond having at least ordinal scale, unidirectionality, and co-directionality with the JPD. Variables may be highly skewed, dependent, and of different types (nominal, categories, ordinal, interval, count, or continuous). With a JPD index, items' dependencies are respected–not just the covariances, and the information in all moments of different orders is preserved. Note that indices need not necessarily designed to refer to a ‘latent dimension’. The Charlson comorbidity indices, Economics and most other indices are such summaries. In observational studies, propensity score [10] is another instance. Propensity score is indeed a dimension-reducing index that reduces several possible confounders into a single score.

In the present paper, we compare the JPD scores with (Confirmatory) Factor Analysis and IRT scorings in two unique datasets. We first present the alternative JPD Index for the 7-item Brief Pain Interface Inventory (BPI-I) [cite] and then construct the JPD index for ordering mental health patients based on 6 administratively collected indicators. For both datasets, we then compare i) the JPD index, ii) factor analytic score when used as an index, and Person’s parameter from the Partial Credit model (PC) [11], the Generalized Partial Credit model (GPC) [12], and the Graded Response model (GRM) [13].

## Background

Dimensionality reduction (DR) of p-variate data to a smaller set of q statistics overcomes computational challenges in data analysis [14–16] and [17]. Several supervised and unsupervised methods, both probabilistic, geometric, or mathematical have been developed. In DR literature, dimension refers to the number of summary statistics constructable from the multivariate data that: 1) are linearly independent and have no overt and hopefully hidden *non-linear* dependency, and 2) satisfy some optimality criteria in preserving a notion of information. In some DR methods, such as factor analysis, composite score, and index construction, it is also desirable that the derived summary or summaries, 3) can be interpreted as quantifying some unmeasurable constructs. Implication of this 2- or 3-step procedure has been the source of ongoing DR research. The lack of unique optimality criteria has been a source of subjective ad hoc decisions in DR procedures. Also, the impossibility of ensuring the nonexistence of nonlinear dependence has resulted in more restricted research formulation, e.g., mostly restricting to linear DR. The current study is about constructing an index, a single composite score, possibly non-linear, that can replace the indicators.

A quick review of DR methods has been reported by [18]. Textbooks on dimensionality reduction (DR) provide a more detailed account of the theoretical basis of these methods [19–21].

When DR results in a single statistic with codirectionality property, the statistic can be used as an index. There is a vast literature on classical index construction independent of DR research [See for example: [22–24]. Also, The United Nations Statistics Division recommendations for index construction [25].

Another conceptually similar problem is the construction of composite scores, in the scoring of instruments and tests, where one is to reduce several item responses to a single statistic for assessment purposes. The scoring methods in Classical Test Theory [26], various Factor Analysis formulations, provided some notion of unidimensionality of factor structure holds, and the Person’s parameter of an appropriate Rasch and more general IRT models [27, 28], all also can be used to construct indices, although these methods have broader agenda then index construction. The DR property of FA and IRT, especially when some notion of unidimenionality holds, is the most relevant feature of the methods to index construction. For completeness, we have briefly introduced these two methodologies in Additional file 1. In the following we introduce the data and the detailed implementation of the methods that will be used in the two examples.

## Materials and methods

### Design

The cross- sectional baseline data collected are from two samples: the *E*ffects of *P*rescription *O*pioid *Ch*anges (EPOCH) [29] and Long *T*erm Outcomes in Veterans *R*equesting *A C*ompensa*tion* (TRACTION) [30] studies. Analyses for EPOCH were post-hoc and pre-planned for TRACTION. The Minneapolis VA Health Care System’s Internal Review Board for Human Studies reviewed and approved the studies’ protocol (#4495-B and #4586-A).

### Participants

EPOCH survey cohort participants were 9,253 randomly selected Veterans with chronic pain who were receiving long-term opioid treatment as part of their management plan who completed a baseline survey. Of baseline survey responders, 364 did not complete all the pain interference inventory items and were excluded from these analyses [29]. Participants from TRACTION were 960 representatively sampled, gender-stratified Veterans who had served during Operations Enduring Freedom, Iraqi Freedom, and New Dawn and had pending VA disability claims for posttraumatic stress disorder Forty-eight TRACTION members did not use any VA health care during the study and are excluded from these analyses [30].

### Data sources

Data sources were self-report survey (EPOCH) and administrative data from the VA Corporate Data Warehouse data (TRACTION). Data were collected between November 2015 and April 2017.

## Measures

### Pain interference

The short-form Brief Pain Inventory (BPI) [31] is widely used to assess clinical pain [32, 33]. Factor analysis points to two latent pain dimensions: “severity,” with four items, and “interference,” with seven items. BPI interference items have 11 response options scored from 0 to 10, with 0 = “Does not interfere” and 10 = “Completely interferes.” Analyses here are confined to the 7 interference items included in the BPI interference (BPI-I) short scale that was collected from the baseline EPOCH questionnaire. Items one to seven are concerned with 1: mood, 2: work, 3: general activity, 4: walking, 5: relationships, 6: enjoyment of life, and 7: sleep.

### Psychiatric severity

The Manifestations of Psychiatric Severity Index (MoPSI) is a measure of psychiatric severity we developed to assess non-response bias in a randomized trial of survey methods [34]. MoPSI is comprised of 6 variables: the number of emergency department visits, psychiatric hospitalizations, and mental health visits TRACTION participants made in a 6-month period as well any ICD-9 and ICD-10-CM codes pertaining to self-harm behaviors, including suicidality; any diagnosis of substance use; and any diagnosis of alcohol use in the 180 days prior to survey. The rationale for selecting these 6 variables is reported elsewhere [34].

Note that MoPSI items do not lend themselves well to traditional psychometric covariance decomposition techniques, such as factor analysis, because there is a mix of data types (e.g., counts and dichotomous) with extreme skewness. Also, local independence of indicators, needed in IRT modeling does not hold across some variables. For example, even for subjects with a fixed person’s parameter value, one expects self-harm behaviors to trigger a more intense health care response (e.g., more clinic visits, emergency department visits, or hospitalizations) than for someone without self-harm behaviors and the same fixed person parameter. See the details below.

### Analysis

We used R 4.2.1 and SAS version 9.4 for all analyses.

## BPI-I scoring

### A. Variance–covariance based scoring

Per published recommendations [31] based on classical factor analysis, scores for the BPI Interference scale were obtained by averaging participants’ responses across the 7 items. The factor structure of the BPI-I has been thoroughly explored in prior studies, e.g., [32, 33]. Alphas showed good internal consistency was 0.89 to 0.92 for the seven interference items [32]. A reported value for test–retest reliability was 0.97 for Pain Interference in a study on 109 patients [35]. However, we reexamined the psychometric properties of the brief BPI-I for our cohort.

In the following we are using Pearson correlation matrix for reliability, suitability and dimensionality identification, and our final confirmatory factor analysis and construct validity assessment. A sample of 8,889 patients who had used opioid for pain reduction for at least last 2 months prior to the data collection endorsed the BPI-I. Cronbach Alpha for BPI-I scale was 0.91 and after deleting each item it stayed within 0.89 to 0.92 showing acceptable reliability for the items.

The Bartlett's test of sphericity was significant (χ^2^(21) = 41,465.36, *p* < 0.001). The overall KMO value for our data was 0.897. Both criteria suggesting that the data are *probably suitable* for factor analysis. To assess the dimensionality, Comparison Data method, Lower bound of RMSEA 90% confidence interval and Akaike Information Criterion, all suggested 3 latent factors, while Hull method with CAF and Parallel Analysis with SMC identified 2 factors. However, the majority of other criteria: Empirical Kaiser criterion, Hull method with CFI, and with RMSEA, Kaiser-Guttman criterion with PCA, and with SMC, and the more common Parallel Analysis with PCA, all suggested unidimensionality. The fit measures of the confirmatory factor analysis, assuming a single construct, were χ^2^(14) = 4800.91, *p* < 0.001, CFI = 0.885, with RMSEA = 0.196 and SRMR = 0.056. The CFI analyzes model fit by examining the discrepancy between the data and the hypothesized model. SRMR is an absolute measure of fit and is defined as the standardized difference between the observed correlation and the predicted correlation. Also, RMSEA, the average of the residual variance and covariance. With these criteria, unidimensional model is marginally acceptable. The widely accepted BPI-I scoring is based on the total sum of the items and assumes a unidimensional construct for pain interference. Note that only 66% of total variance was explained by this factor. Factor loadings of CFA for BPI-I1 through BPI-I7 were 1.83, 2.12, 1.87, 1.93, 2.32, 1.84, and 2.22, respectively. The Pearson correlation between factor scores and the total sum was 0.996, supporting the practice of using sum score as the factor score.

### B. IRT modeling of the BPI-I items

In Item Response Theory the estimated subjects’ ability, can be used to rank order the subjects. Here we will refer to the “ability” as “vulnerability” (${\theta }_{v})$ namely, vulnerability to pain-related functionality indicators. We used Masters’ [11] Partial Credit models(PC), Generalized PC (GPC), and Graded Response Models(GRM) to estimate participants’ vulnerabilities. With (Generalized) Partial Credit models and Graded Response Model, indicators may be recorded as categorical, ordinal, or even continuous [36, 37]. For the present analysis, we assumed each BPI-I item was an ordered response with 11-categories (0–10).

Specifically, the probability that person $v$ choses category $h=\text{0,1},...10$ of ith BPI-I item is defined as:

2 $$P\left({X}_{vih}=h\right)=\frac{\text{exp}\left[h{\theta }_{v}+{\beta }_{ih}\right]}{\sum_{l=0}^{l=10}\text{exp}\left[l{\theta }_{v}+{\beta }_{il}\right]}$$

If assumptions of Item Response Theory hold, we would expect higher vulnerability to be associated with greater probability of endorsing higher or severer response options in all seven items. To investigate these, we used ICC and Items maps. The item characteristic curves (ICC) for each BPI-I item, relating the probability of endorsing any value between 0 to 10 for each question against a person’s estimated vulnerability. Values with steep ICC indicate good discriminant power. Person-item map can be used to examine the association between participants’ vulnerabilities and their probability of endorsing higher (severer) options for each BPI-I item. The R packages mirt: A Multidimensional Item Response Theory Package [38] and eRm: Extended Rasch Modeling [39] and psych package: Procedures for Psychological, Psychometric, and Personality Research [40] were used for the analyses.

### C. JPD method for BPI-I items

The joint density or probability function by its definition is an expression of frequencies in the presence of dependencies. In technical sense it is radon-Nikodym derivative of the underlying probability model usually with respect to Lebesgue or counting measure. As detailed in Additional File 2, Appendix, to specify the 7-dimensional joint probability distribution for the BPI-I, we used all 7! (= 5040) permutations of the chain rule factorization:  

3 $$f\left(x_1,x_2,\dots,x_7\right)=f\left(x_1\right)f\left(x_2\vert x_1\right)\dots f\left(x_7\vert x_2,\dots,x_6\right)$$

where *x*_1_ through *x*_7_ corresponds to the 7 BPI-I items.

To model the conditional dependencies, we assumed each conditional model $f\left({x}_{i}|{x}_{{l}_{1}},\dots ,{x}_{{i}_{k}}\right)$ had a two-parameter Gamma distribution whose mean, $\mu =E\left({X}_{i}|{x}_{{l}_{1}},\dots ,{x}_{{i}_{k}}\right)=\sum {\beta }_{j}{x}_{{i}_{j}}$, was expressed as a linear function of all the conditioning variables and $b=\frac{1}{s}$, where $b$ is the Gamma distribution’s rate parameter, and $s,$ the scale parameter. We used the log transform of $f\left({x}_{1}, {x}_{2},\dots ,{x}_{7}\right)$ and then ML estimation of the parameters via generalized linear model (see Additional File 1 for code) estimates the log density= $\widehat{b}\left(\frac{{-x}_{i}}{\widehat{\mu }}-\text{log}\left(\widehat{\mu }\right)\right)-\left(\widehat{b}-1\right)\text{log}\left({x}_{i}\right)$ for each conditional model $f\left({x}_{i}|{x}_{{l}_{1}},\dots ,{x}_{{i}_{k}}\right).$ The average of the 5040 permutations;

4 $$log\left(f({x}_{1}, {x}_{2}, . . ., {x}_{7}\right))= \sum_{7!}\frac{\text{log}\left(f\left({x}_{{i}_{1}}, {x}_{{i}_{2}}, . . ., {x}_{{i}_{7}}\right)\right)}{7!}$$

Or any monotone transformation of that is the participant’s BPI-I score. Note that each of the 5040 conditional specifications provide estimates of the same JPD. The variations between the estimates:

$$\sum_{7!}\frac{{(\text{log}\left(f\left({x}_{{i}_{1}}, {x}_{{i}_{2}}, . . ., {x}_{{i}_{7}}\right)-log\left(f({x}_{1}, {x}_{2}, . . ., {x}_{7}\right))\right)}^{2}}{7!-1}$$

provides a measure of accuracy of the estimators. To assess the sampling error of each person’s JPD estimate, we used 50 bootstrapped samples (with replacement) constructed within subject standard deviation of the bootstrapped JPD estimates. The estimated standard errors for 8889 subjects in the pain study were between 0.19 to 1.89 when JPD was scaled to 0–10. For each subject our estimation method produces 5040(7!) JPD estimates, the variation between these estimates coming from alternative specifications provides a measure of accuracy of reported JPD which is an average of these scores. The between specifications standard deviations for each subject BPI_I JPD scores were between 0 and 0.252. These shows a rather robust JPD subjects’ estimates with respect to the conditional factorization of the joint pdf.

## MoPSI Scoring

### A. Variance–covariance based scoring

To use the factor analysis method, one needs to construct a matrix of “correlations” between the indicators (items). Three items (Mental health clinic, hospital, and emergency room visits) are count data where their larger values indicate more severe cases of mental health. Substance use (SUD), suicidality, and ETOH are ordered binary indicators, where (1) indicates a worse case compared to (0). The first challenge in EFA and subsequent confirmatory modeling is to decide how to quantify their associations (correlations). Spearman correlations (and several other measures of monotone dependence) may be used to construct a symmetric matrix. Instead of modeling dependence via ranks, one may construct a matrix of heterogeneous correlations consisting of Pearson product-moment correlations between numeric variables, polyserial correlations between numeric and ordinal variables, and Polychoric correlations between ordinal variables. Alternatively, we may ordinally categorize all indicators and use Polychoric correlation uniformly for all the *p*(p + 1)/2 pairs of variables. Hopefully, these symmetric matrices will be positive definite to qualify as a correlation matrix. The existence of multitude of possibilities has consequences in identifying the dimensionality of latent factor structure and especially the quality of the unidimensional index.

Specifically, here we explored the Spearman correlation (Scor), a Polychoric correlation matrix (Ocor), assuming the number of hospitalizations (0–4), and the number of emergency visits (0–3) being ordinal and, a matrix (Mcor) of mixture of correlations, where for non-categorical pairs Spearman’s rho, tetrachoric correlations for binary pairs, and Glass rank biserial for binary and number of visits are used. The Mcor matrix, though, is non-singular: it is not positive definite (the smallest eigenvalue is negative). This phenomenon often happens with these remedies when one tries factor analytic methods on data with varying scales. In the present example, this required ad hoc smoothing when performing dimension identification [41].

To develop confirmatory factor scores, we first identified if each of these correlations is suitable for factor extraction. The table below summarizes the results (Table 1).

**Table 1:** Suitability of data for FA

Test/Correlation	Spearman(Scor)	Ordinal(Ocor)	Mixed (Mcor)
Bartlett's test of sphericity	χ^2^(15) = 1036.99, *p* < .001: Suitable	χ^2^(15) = 1025.45, *p* < .001: Suitable	χ^2^(15) = 21,990.03, *p* < .001: Suitable
Kaiser–Meyer–Olkin criterion (KMO)	0.744: Suitable (middling)	0.765: Suitable (middling)	0.203: Not suitable (unacceptable)

As it is seen, suitability of data for dimensionality identification depends on the subjective decisions involved in quantifying dependence. Unfortunately, there is also no unique optimal criteria for identifying the dimensionality. Table below lists the number of latent dimensions suggested by some more common criteria (Table 2).

**Table 2:** Number of latent dimensions

Test/Criteria	Scor	Ocor	Mcor
Empirical Kaiser criterion	1	1	2
Hull method with CAF	1	1	0
Hull method with CFI	2	2	1
Hull method with RMSEA	2	2	1
Kaiser-Guttman criterion with PCA	2	2	2
Kaiser-Guttman criterion with SMC	1	1	2
Parallel analysis with PCA	2	1	2

*SpCor *Spearman Correlation*, Ocor *Polychoric Correlations*, Mcor *mixture of Correlations

Next, for each of the three correlation matrices, we used EFA model averaging to estimate the one- and two-dimensional factors. The averaging was over estimation methods (Principal Axis, MLE and unweighted least squares), initial values and criterion type. Averaging performed via mean, with no trimming, across 108 EFAs for 2-factor model and 9 for one-factor model. The error rate was zero; all the solutions converged except for the mixed correlation matrix, where only 78% of alternative scenarios converged. All the solutions were admissible with no Heywood cases [cite] for all three matrices.

For reliability of the unidimensional CFA scores, assuming the ordinal scales, after categorizing number of mental health visits into eight categories, with Polychoric correlations, have ordinal alpha [42] of 0.923. The Omega, that is the ratio of variance of loadings times the latent factor over observed variance, was 0.749, however, the modified Omega (denominator variance being the CFA-implied variance) was 0.753. For developing an insight about construct validity of the unidimensional and two factor CFA models, fit indices are listed in the table below: [Table 3].

**Table 3:** Fitness measures for Psych Severity Indicators (M (SD) [Min; Max])

Model	Spearman	Polychoric	Mixture
One factor df = 9	χ^2^: 122.47 (36.00) [80.91; 143.26]	104.54 (34.83) [64.33; 124.65]	297.46 ( NA) [297.46; 297.46]
	*p*: .000 (.000) [.000; .000]	.000 (.000) [.000; .000]	.000 (NA) [.000; .000]
	CFI: .92 (.03) [.90; .95]	.93 (.02) [.92; .96]	0.92 (NA) [.92; .92]
	RMSEA: .12 (.02) [.09; .13]	0.11 (.02) [.08; .12]	0 .19 (NA) [.19; .19]
	AIC: 104.47 (36.00) [62.91; 125.26]	86.54 (34.83) [46.33; 106.65]	279.46 (00) [279.46; 279.46]
	BIC: 61.13 (36.00) [19.57; 81.92]	43.20 (34.83) [ 2.98; 63.31]	236.12 ( 00) [236.12; 236.12]
	CAF: .42 (.01) [.41; .43]	0.45 (.00) [.45; .45]	.44 (.00) [.44; .44]
Two factors df = 4	χ^2^: 4.23(1.75)[1.79; 5.45]	11.80 (4.87) [ 5.01; 15.20]	11,680.47 (8349.26) [37.98; 17,501.71]
	*p*-value: 0.421(0.254) [.244; .775]	0.098 (0.135) [0.004; 0.286]	0.000 (.000) [.000; .000]
	CFI:1.00 (.00)[1.00; 1.00]	0.99 (.00) [.99; 1.00]	0.33 (.47) [0.00; 0.99]
	RMSEA: 0.01(.01) [.00; .02]	0.04 (.02) [.02; 0.06]	0.70 (.43) [0.10; 1.00]
	AIC:-3.77(1.75)[-6.21;-2.55]	3.80 ( 4.87) [-2.99; 7.20]	11,672.47 (8349.26) [29.98; 17,493.71]
	BIC:-23.03(1.75)[-25.48; -21.81]	-15.46 ( 4.87) [-22.25; -12.06]	11,653.20 (8349.26) [10.71; 17,474.45]
	CAF: 0.51(.00)[.51;.52]	0.49 (.00) [0.49; 0.50]	0.45 (.01) [0.42; 0.45]

For one dimensional model, all indices indicate that the polychoric correlation structure fits better than the Spearman and the mixture of correlations. As always, the two-dimensional model indices indicate closer fit. For our comparisons we will use the factor scores of one-factor model in the corresponding confirmatory factor analysis.

### B. Item response modeling of MoPSI items

We will use PC, GPC and GR models to estimate the corresponding participants’ vulnerability. We treated the number of mental health visits, emergency department visits, and hospitalizations as ordinal variables. However, to assess the quality of these indices (estimated Person’s paramter: vulnerabilities) a person-item map was graphed. This visually showed codirectionality between participants’ vulnerabilities (the indices) and their probability of endorsing higher (severer) options for each MoPSI items.

### C. JPD method for the MoPSI

Consistent with our approach to estimating the BPI-I’s joint probability density, detailed above, we specified the MoPSI’s 6-dimensional joint probability distribution by using all 6! (= 720) permutations of the chain rule factorization:

5 $$f\left(x_1,x_2,\dots,x_6\right)=f\left(x_1\right)f\left(x_2\vert x_1\right)\dots f\left(x_6\vert x_2,\dots,x_5\right)$$

where *x*_1_ = number of mental health clinic visits, x_2_ = number of emergency department visits for mental health concerns, *x*_3_ = number of psychiatric hospitalizations, *x*_4_ = self-harm diagnoses, *x*_5_ = substance use diagnoses, and *x*_6 =_ alcohol use diagnoses.

To model the conditional dependencies, we used logistic regression for the 3 binary diagnosis variables (self-harm, alcohol use, and substance use) and linear regression for the 3 count variables (number of mental health clinic visits, emergency department visits, and hospitalizations). Based on Bayesian information criterion (BIC) and Akaike information criterion (AIC), we found that the Poisson-Inverse Gaussian (PIG) distribution for the count variables had better fit indices than the Poisson and Negative Binomial and their zero-inflated versions. Each permutation of the factorization yields 720 new conditionally specified models that capture slightly different dependency structures. These terms each provide a single estimate of the same joint model, $f\left({x}_{1}, {x}_{2},\dots ,{x}_{6}\right)$ or, after taking the log monotone transformation, of $f\left({x}_{1}, {x}_{2},\dots ,{x}_{6}\right)$. Therefore, as in (3), to utilize all the information captured by the 6! models, we used the average of 720 estimates of $log\left(f({x}_{1}, {x}_{2}, . . ., {x}_{6}\right)),$ asthe participant’s JPD MoPSI score. To estimate standard error of the estimated score per person, we calculated standard deviations of 40 bootstrapped estimates of JPDs for each person. The bootstrapped estimated sampling standard errors for all subjects were between (0.001, 2.433) with mean boosted SE for all subjects being 0.792. The standard deviations of each person estimated JPD between 720 specifications, for all subjects were between 0.504 and 3.382, with an average SD of 1.106.

Note that this information probably shows that we might not need to run all the possible permutations. A random sample of permutations might suffice.

The R code used to estimate the joint probability density of the MoPSI components is included in Additional File 1.

### Unidirectionality and co-directionality

Assumptions of unidirectionality and co-directionality are the minimum required to conceptually label any summary as an index. Classical measures of monotone dependencies, such as rank correlations, may be used to assess for the strength of unidirectionality and should be positive. To account for skew in the BPI-I items, we used Spearman’s rho and Kendall’s tau to examine the pair-wise rank correlations of BPI-I items and to compare the correlation between each item and the 3 composite BPI-I scores obtained via standard scoring, Item Response Theory modeling, or JPD method. For the MoPSI, we examined pair-wise rank correlations using Spearman’s rho for non-categorical pairs, tetrachoric correlations for pairs of binaries, and the Glass rank biserial for binary/continuous pairs.

For co-directionality, subjects’ rankings provided by the summary or composite score should agree with each indicator’s ranking. The strength of this monotone agreement may also be assessed by rank correlations and should be positive. We used Spearman’s rank correlation and Kendall’s tau to correlate individual items to the MoPSI summary scores obtained through IRT modeling and the JPD method.

### Comparing scoring approaches

We used simple scatterplots and Spearman’s rank correlation to compare the three BPI-I scoring approaches and to compare the two MoPSI scoring approaches, where 0 = no monotonic relationship and 1.00 indicates perfect, positive monotone relationship. Note that ranks stay invariant with respect to monotone transformations.

To more formally compare the approaches’ informativeness, we used maximum likelihood to estimate Shannon’s entropy [43]. Lower entropy values indicate that more information is contained within a summary or composite score. BIC and AIC indices indicated that a Weibull distribution was a better fit for the BPI-I and a two-parameter Gamma distribution, for the MoPSI. Shannon’s entropy $E$ for Weibull distributions is calculated as follows:

6 $$E=\gamma \left(1-\frac{1}{k}\right)+\text{ln}\left(\frac{k}{l}\right)+1$$

where $\gamma$ is Euler’s constant (approximately 0.57712), $k$ = shape and $l$ = scale. “Shape” refers to the actual shape or form of the distribution (e.g., is it peaked, rounded, flat?) and “scale” refers to the distribution’s dispersion. For two-parameter Gamma distribution, the entropy is:

7 $$E=a-\text{log}\left(b\right)+\text{log}\left(\Gamma\left(a\right)\right)+\left(1-a\right)*\frac{\text{d}\text{log}\left(\Gamma\left(a\right)\right)}{\text{d}a}$$

where $a$ = shape and $b$ = rate, where the rate is the inverse of scale.

For each BPI-I and MoPSI scoring approach, we also examined the number of distinct values (person-scores) returned by each scoring method. A higher number of distinct values signifies greater granularity and informativeness.

### Outcomes

The main outcome is development of an unsupervised dimension reduction method based on joint pdf and noticing that the joint probability function under some plausible conditions may be used as an index. This composite score is the most informative unidimensional reduction of multivariate data. The BPI-I and MoPSI JPD-scores were developed as two applications of these results.

### Power

This research does not test any hypothesis and hence power analysis is not relevant.

## Results

Table 4 shows the summary statistics of the different BPI-I and MoPSI scoring approaches.

**Table 4:** Distribution statistics from scoring methods of the brief pain inventory-interference scale and scoring methods of the manifestations of psychiatric severity index

Scoring Method	Mean	SD	Median	1st Quartile	3rd Quartile
**BPI-I**					
Standard	6.514	2.114	6.857	4.411	8.143
CFA Score	5.740	1.780	5.766	4.571	6.922
PC Model	5.535	1.764	5.488	4.341	6.651
GPC Model	5.555	1.776	5.473	4.383	6.643
GR Model	5.78300	1.745	5.816	4.658	6.935
JPD	9.082	1.152	9.363	8.926	9.652
**MoPSI**					
CFA(Spearman)	0.763	1.079	0.279	0.000	1.118
CFA(Polychoric)	1.251	1.500	0.499	0.000	1.999
CFA(Mixture)	2.534	2.725	1.130	0.000	4.522
PC Model	2,404	2.142	1.645	0.000	3.967
GPC Model	2.107	2.261	1.321	0.000	3.488
GR Model	2.193	2.192	1.891	0.000	3.481
JPD	0.428	1.202	0.014	0.000	0.128

*SD* Standard Deviation, *IQR* Interquartile Range, *BPI-I* Brief Pain Inventory Interference scale, *MoPSI* Manifestations of Psychiatric Severity Index, *JPD* Joint Probability Density

Table 5 shows the inter-item correlations of the seven BPI-I items and the item-to-score rank-correlations. As can be seen in the Table, all seven items are positively correlated and hence, unidirectional. Rank-correlations of the 7 items with the summary (composite) scores range from 0.605 to 0.897, suggesting moderate to strong co-directionality of all scorings with the items. The high rank-correlation between the standard, CFA, IRT scores, and JPD summary indices for BPI-I scoring suggests the JPD method produces scores with ranks highly concordant with the other methods. This demonstrates that JPD scoring encapsulates all the information captured by covariance structure decomposition (CFA), Persons’ vulnerability scores in IRT sub-models restricted by local independence assumption. Note that high rank correlations between IRT scorings and CFA confirms that the broader IRT models agree with CFA, provided the covariance matrix is the only set of parameters identifying the joint probability distribution. For centered set of indicators with a spherically symmetric distribution, like a multivariate normal distribution, CFA scoring then is equivalent to an appropriate IRT and more generally, the JPD scoring.

**Table 5:** Spearman correlations between brief pain inventory interference items and composite scores

	BPI-I Items							Composite Scores					
	1	2	3	4	5	6	7	Sum	GR	GPC	PC	CFA	JPD
1	1.00	0.61	0.64	0.77	0.58	0.57	0.65	0.833	0.865	0.839	0.814	0.855	0.698
2	0.61	1.00	0.46	0.55	0.74	0.57	0.67	0.821	0.783	0.805	0.813	0.896	0.639
3	0.64	0.46	1.00	0.74	0.47	0.46	0.55	0.751	0.735	0.713	0.723	0.740	0.608
4	0.77	0.55	0.74	1.00	0.56	0.55	0.64	0.834	0.850	0.820	0.803	0.857	0.700
5	0.58	0.74	0.47	0.56	1.00	0.58	0.68	0.827	0.780	0.807	0.820	0.897	0.605
6	0.57	0.57	0.46	0.55	0.58	1.00	0.62	0.766	0.717	0.718	0.718	0.712	0.623
7	0.65	0.67	0.55	0.64	0.68	0.62	1.00	0.852	0.832	0.829	0.819	0.825	0.700
Sum	0.833	0.821	0.751	0.834	0.827	0.766	0.852	1	0.975	0.973	0.974	0.980	0.802
GR	0.865	0.783	0.735	0.850	0.780	0.717	0.832	0.975	1	0.990	0.985	0.996	0.745
GPC	0.839	0.805	0.713	0.820	0.807	0.718	0.829	0.973	0.990	1	0.997	0.995	0.744
PC	0.814	0.813	0.723	0.803	0.820	0.718	0.819	0.974	0.985	0.997	1	0.991	0.744
CFA	0.855	0.896	0.740	0.857	0.897	0.712	0.825	0.980	0.996	0.995	0.991	1	0.762
JPD	0.698	0.639	0.608	0.700	0.605	0.623	0.700	0.802	0.745	0.744	0.744	0.762	1

*BPI-I* Brief Pain Inventory-Interference scale, *JPD* Joint Probability Density, *GR* Graded Response Model, *GPC* Generalized Partial Credit Model, *PC* Partial Credit Model, *CFA* Confirmatory Factor Analysis, *Sum* Sum of item’s scores (The established BPI-I score)

**Fig. 1:**
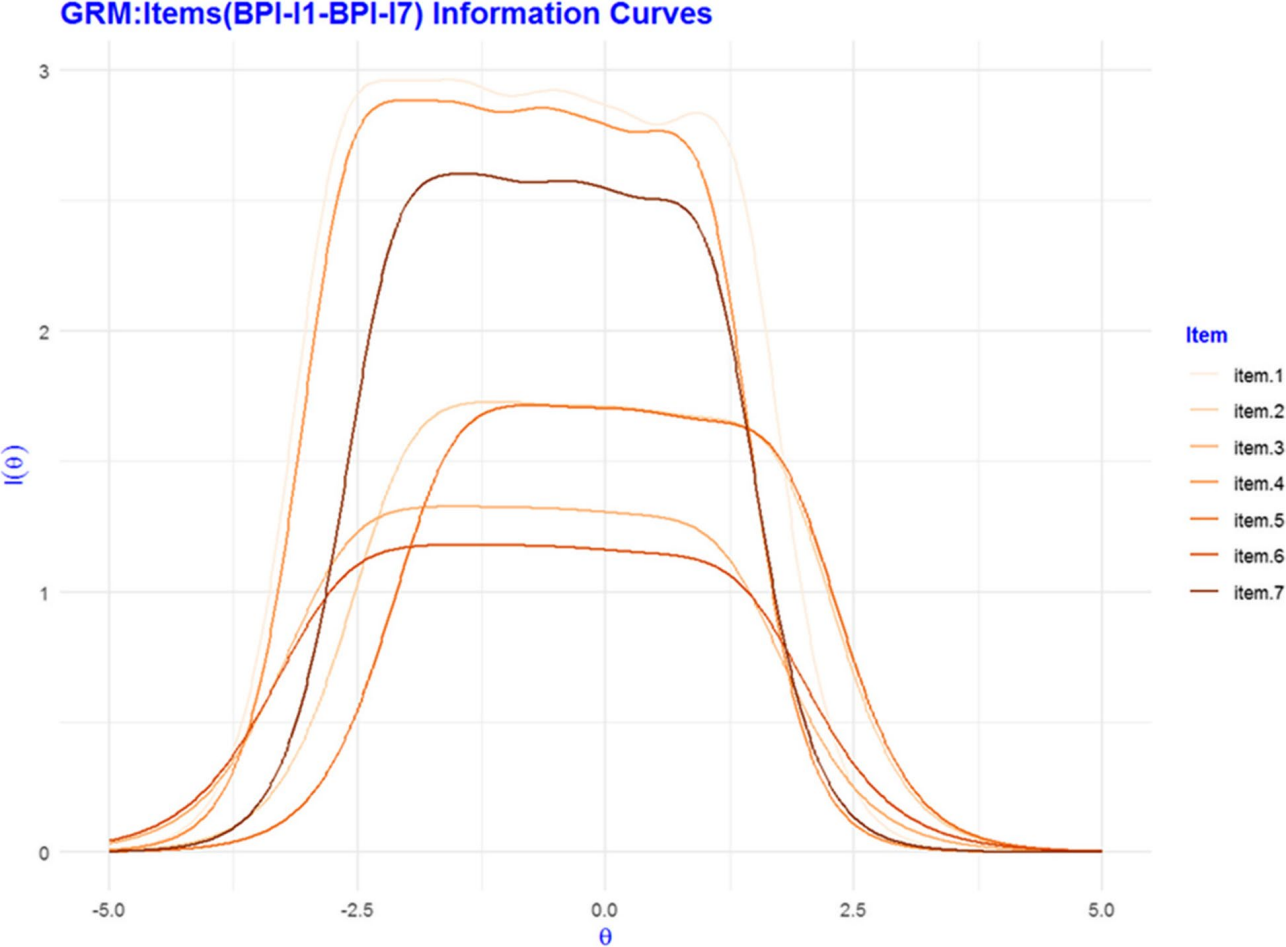
Item characteristic curves for the 7 Brief Pain Inventory Interference items. BPI-I = Brief Pain Inventory Interference scale

Figure 1 shows the BPI-I’s 7 items’ information curves for Graded Response model. As expected, the items are more informative (Fisher information) about participants with middle size vulnerabilities. The items specially items 1 (mood), 4 (walking) and), 7(sleep) are more discriminating items for these vulnerabilities Generalized Partial Credit and Partial Credit models’ information curves, which we have not reported here, demonstrated similar patterns.

**Fig. 2:**
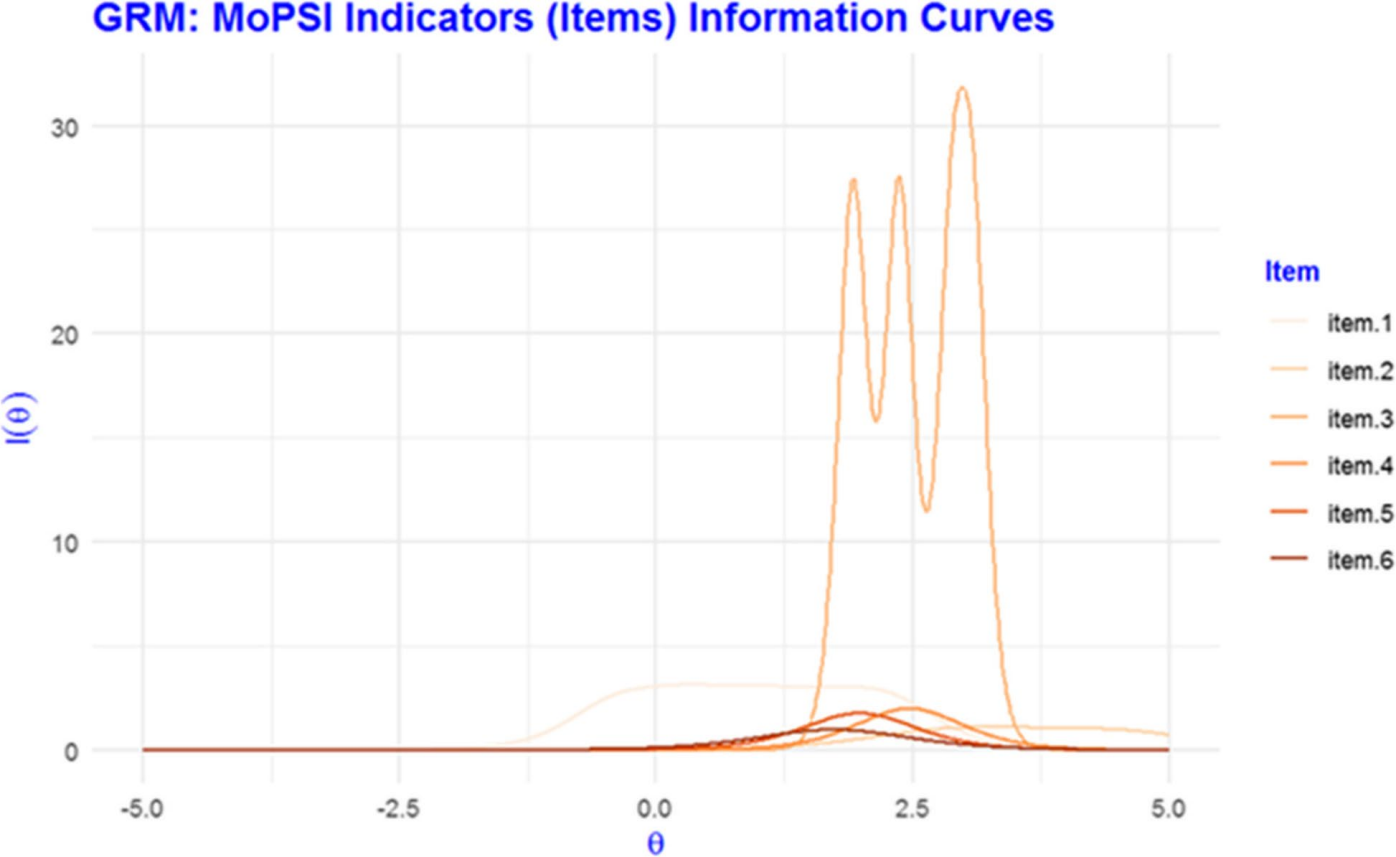
Person-Item map for the 7 Brief Pain Inventory Interference items. Solid circles present the spread of item difficulties and hollow circles, response thresholds across the latent trait (vulnerability). Items are sorted along the y-axis by difficulty. Vul. Dist. = Vulnerability Distribution

Figure 2 depicts items’ information curves for MoPSI indicators. Number of mental health Clinic visits (item 1) provides information over a wider range, including the less sever patients, while number of emergency department visits for mental health concerns (item 2) is more informative, almost non-overlapping about more severe cases (higher Person’s values). Number of psychiatric hospitalizations is the most informative (item 3) to discriminate between subjects with mainly above average severity. The item shows an erratic behavior, however even in its least informative situations is much more information than all other items (4–6): self-harm, substance, and alcohol use. These items are less-discriminating items provided less information but over a wider range of more severe cases.

Table 6 shows the inter-item correlations of the six MoPSI indicators and the item-to-total-score correlations for the 2 scoring versions. As Table 6 shows, the six indicators are positively correlated and thus unidirectional. Correlations of the indicators with the summary scores are positive, again supporting evidence of co-directionality. The indicators’ higher correlations with the JPD method compared to the IRT method suggests that the JPD method generally ranks patients closer to their observed indicators’ rankings. As with the BPI-I, there is a high rank correlation between scores obtained via alternative models.

**Table 6:** Spearman rank correlations between manifestation of psychiatric severity items and composite scores

	MH Visits	ED Visits	Hospitalization	Self-Harm	Substance use	Alcohol use	Composite Scores				
							GR	GPC	PC	JPD	CFA
**Items**											
MH Visits	1.000	0.445	0.281	0.533	0.407	0.313	0.302	0.302	0.298	0.299	0.993
Ed Visits	0.445	1.000	0.180	0.295	0.200	0.145	0.228	0.226	0.220	0.245	0.212
Hospitalization	0.281	0.180	1.000	0.211	0.296	0.352	0.982	0.987	0.991	0.954	0.299
Self-Harm	0.533	0.295	0.211	1.000	0.255	0.165	0.235	0.232	0.228	0.241	0.229
Substance Use	0.407	0.200	0.296	0.255	1.000	0.394	0.361	0.351	0.338	0.382	0.347
Alcohol Use	0.313	0.145	0.352	0.165	0.394	1.000	0.456	0.441	0.429	0.504	0.400
**Scores**											
GRM	0.302	0.228	0.982	0.235	0.361	0.456	1.000	0.999	0.996	0.991	0.999
GPC	0.302	0.226	0.987	0.232	0.351	0.441	0.999	1.000	0.999	0.986	0.999
PC	0.298	0.220	0.991	0.228	0.338	0.429	0.996	0.999	1.000	0.980	0.999
JPD	0.299	0.245	0.954	0.241	0.382	0.504	0.991	0.986	0.980	1.000	0.976
CFA (Polychoric)	0.993	0.212	0.299	0.229	0.347	0.400	0.999	0.999	0.999	0.976	1.000

*MH* Mental Health, *ED* Emergency Department, *JPD* Joint Probability Density, Spearman Rank correlations

**Fig. 3:**
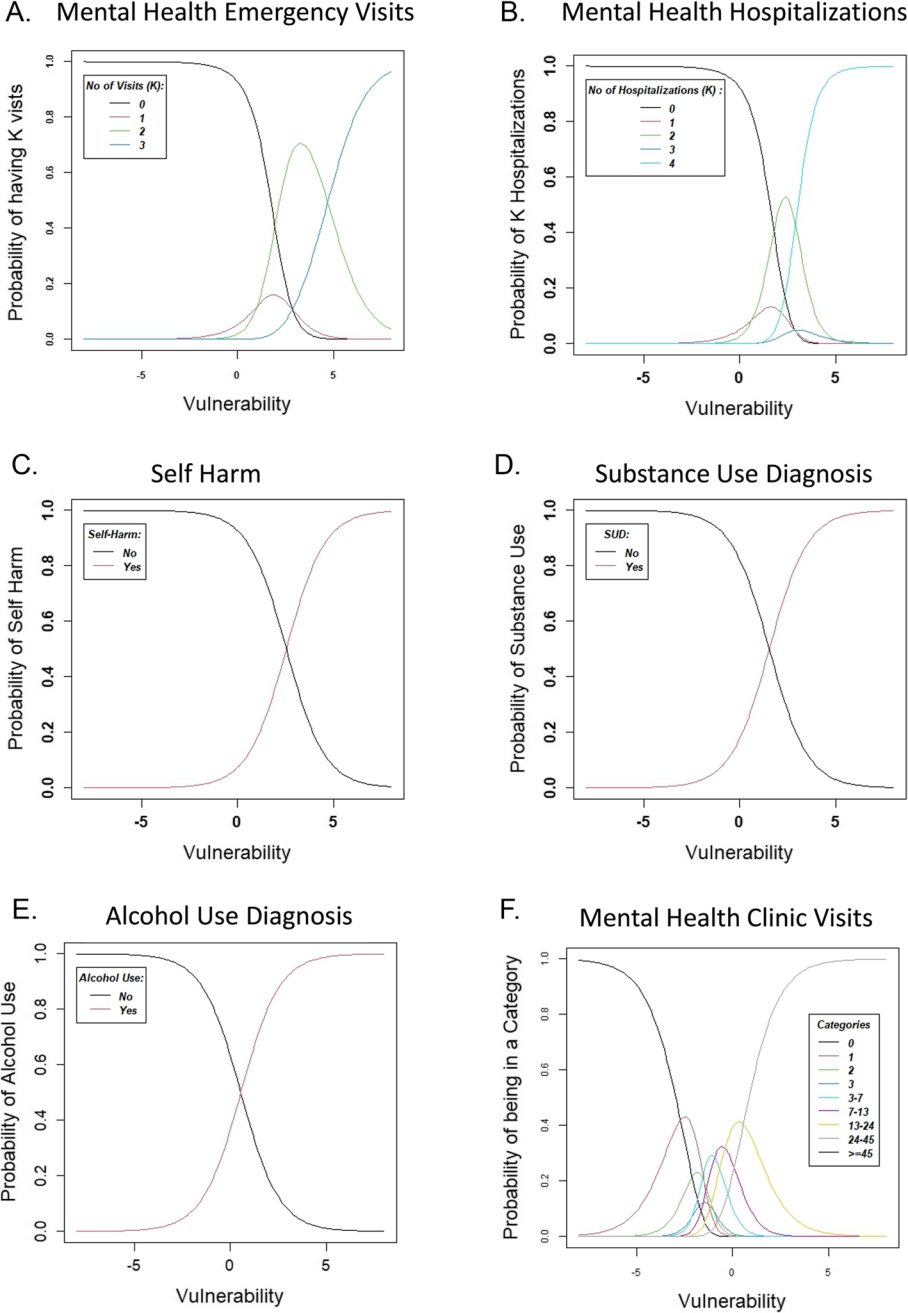
Item characteristic curves for the 6 manifestations of psychiatric severity index items

**Fig. 4:**
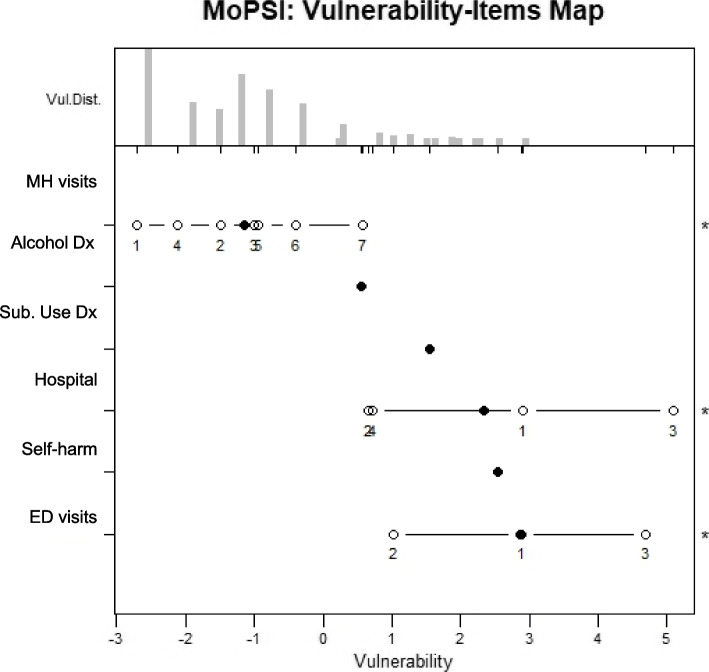
Person-Item map for the 6 Manifestations of Psychiatric Severity Index items. Solid circles present the spread of item difficulties and hollow circles, response thresholds across the latent trait (vulnerability). Items are sorted along the y-axis by difficulty. Vul. Dist. = Vulnerability Distribution. MH = Mental health. Dx = diagnosis. ED = Emergency department

BPI-I is a well-established items and their properties have been reported by several authors. To understand the MoPSI items behavior more closely, Fig. 3 shows the MoPSI’s 6 ICC plots, and Fig. 4, the PC model person-item map. As can be seen from Fig. 3, the 3 ICCs for binary items (suicidality, alcohol use, substance use) show that patient with higher severities have higher probabilities of endorsing them and low sever patients most likely will report no use or thoughts of suicidality are steep and thus can distinguish between more and less vulnerable participants. As Fig. 3, Panels A, B and F show higher values are reported by patients with higher severity while lower values are reported with high probability by less sever patients. ICCs for mental health care visits between 0 and 45 assessed a wider range of vulnerability, which is desirable if one wishes to rank-order individuals along a broad continuum, rather than identify only high (low) vulnerability. This is echoed in Fig. 4, where it shows that the number of mental health clinic visits is most important in covering the lower range of vulnerability, while the number of psychiatric hospitalizations, self-harm diagnoses, and the number of emergency department visits for psychiatric reasons are more important in capturing the higher range of vulnerability.

**Fig. 5:**
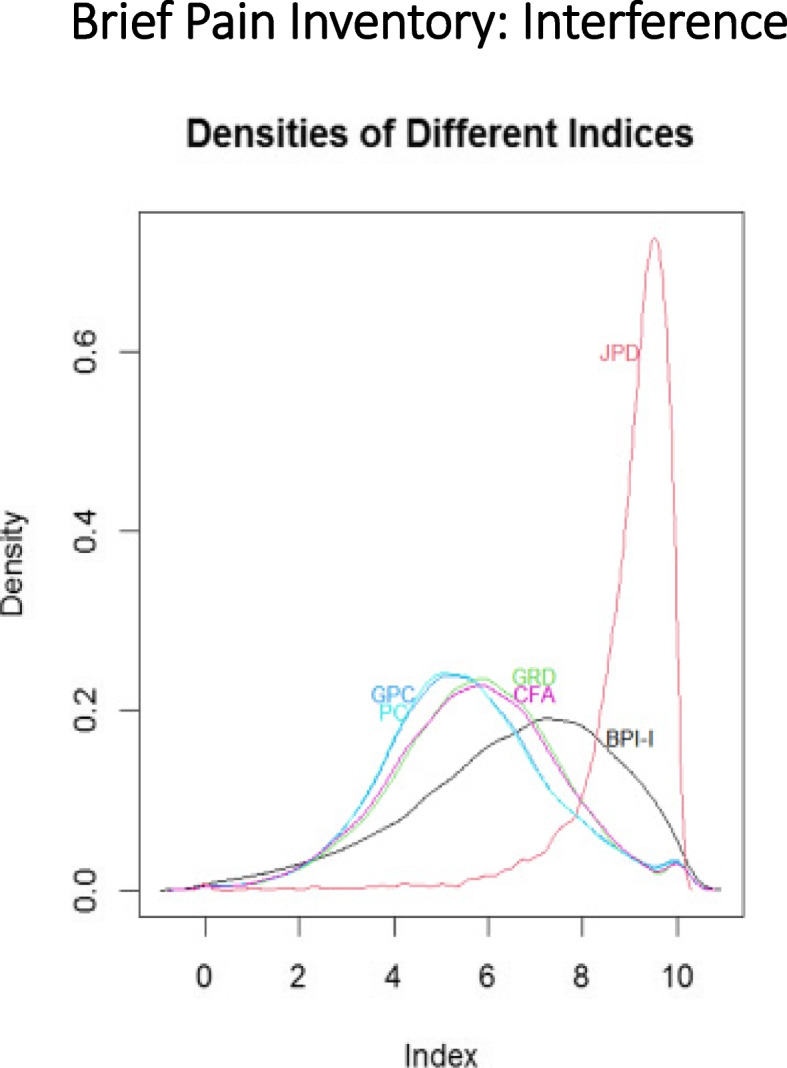
Density plots for brief pain inventory interference scorings. BPI-I = Brief Pain Inventory Interference scale. JPD = joint probability density

**Fig. 6:**
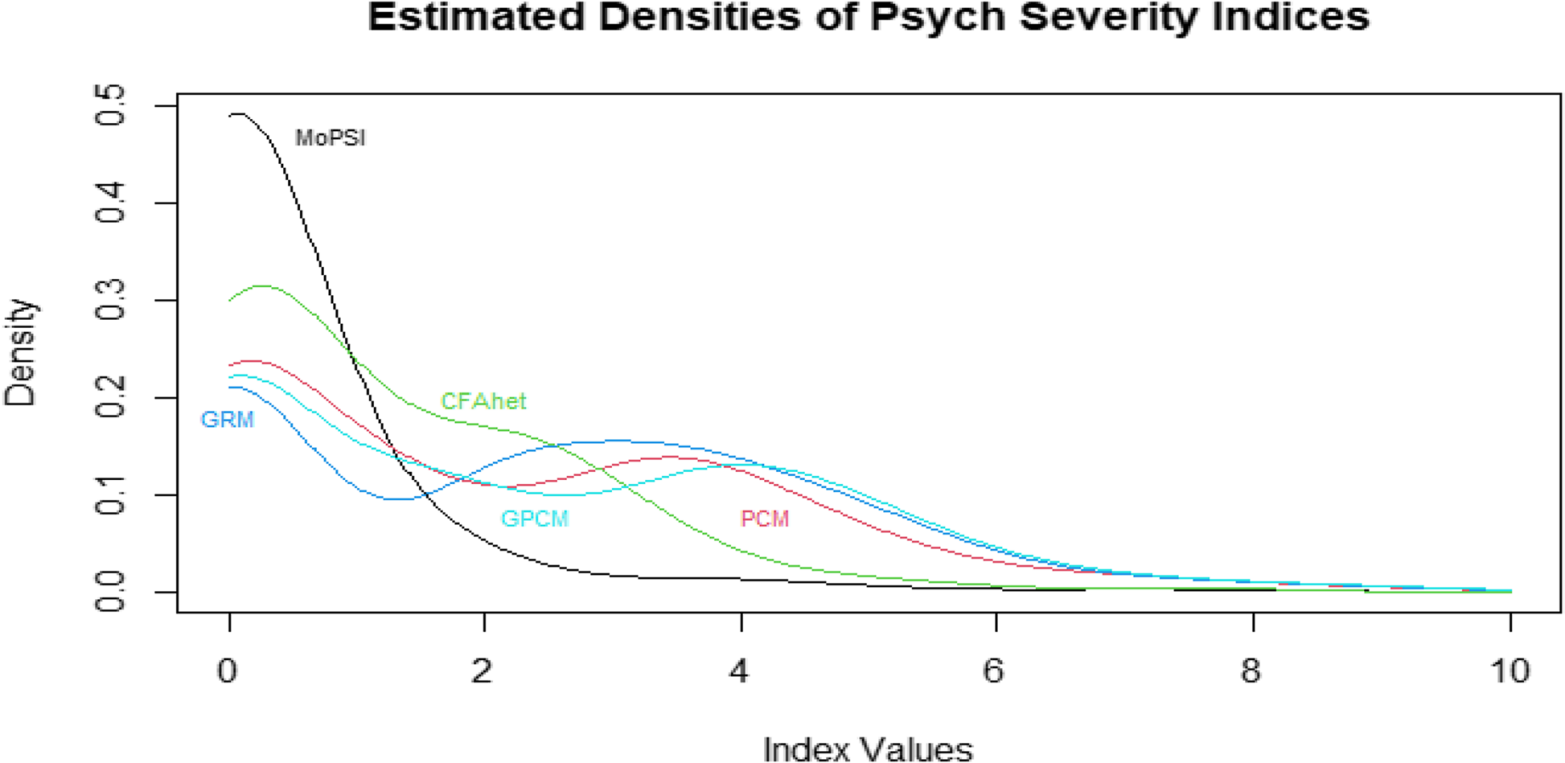
Density plots of manifestations of psychiatric severity index scorings. MoPSI = Manifestations of Psychiatric Severity Index. JPD = joint probability density

Figure 5 shows the density plots of the BPI-I scorings, and Fig. 6, the density plot of the psych severity indices. As can be seen from Fig. 5, the density of the JPD score has highest Pearson Divergence from the uniform distribution over the interval [0,10]. Indeed, the Divergences for BPI-I scoring densities are proportional to: 0.004(BPI-I score), 0.035(JPD),0.007(GRD),0.007(GPC), 0.007(PC) and 0.006(CFA). The same observation holds for the densities of the psych severity scorings:

Shannon’s entropy was consistently lower for the JPD method than for the other methods. Pearson Divergence of the alternaive indices’ densities from the uniform density over the common 0–10 range. The JPD Divergence for BPI-I, is 5 to 8 times bigger than the other indices’ Divergences. The Pearson’s Divergence of distributions of MoPSI scorings from the uniform distribution are proportional to: 0.432, 0.006, 0.034, 0.015, 0.004, 0.006, 0.005, respectively for JPD, PC, CFA (Spearman), CFA (Polychoric), CFA (Mixture of measures of associations), GR and GPC models. As expected, The JPD Divergence is again much larger than the alternatives.

For psych severity scorings, there were no significant difference between the Graded Response model and Generalized Partial Credit model. Both fitted the data. However, all these three models, across different fit indices received a moderate support by data. See Table 7 below for the details:

**Table 7:** IRT Model Comparisons

Scoring	Model	M2	df	*p*	RMSEA	RMSEA_5	RMSEA_95	SRMSR	TLI	CFI
BPI-I	PC	6213.334	20	0	0.187	0.183	0.191	0.095	0.906	0.911
	GPC	4946.723	14	0	0.199	0.194	0.204	0.074	0.893	0.929
	GRM	5803.727	14	0	0.216	0.211	0.220	0.071	0.875	0.916
MoPSI	PC	58.156	14	0.000	0.059	0.044	0.075	0.122	0.966	0.996
	GPC	15.785	9	0.072	0.029	0.000	0.052	0.053	0.992	0.995
	GRM	15.709	9	0.073	0.029	0.000	0.052	0.051	0.992	0.995

The JPD, GRM, GPC, CFA methods for BPI-I returned 7,778 distinct ranks compared to 480 distinct values for the conventional and PC methods. Likewise, the MoPSI’s JPD method produced 121 distinct values (person-scores) compared to 40 values produced by PC modeling. Both Graded Response and Generalized Partial Credit models ranked the subjects into 68 rank-equivalent groups, indicating that the JPD method generally is more granular and can produce finer separations in rank-order.

## Discussion

**Fig. 7:**
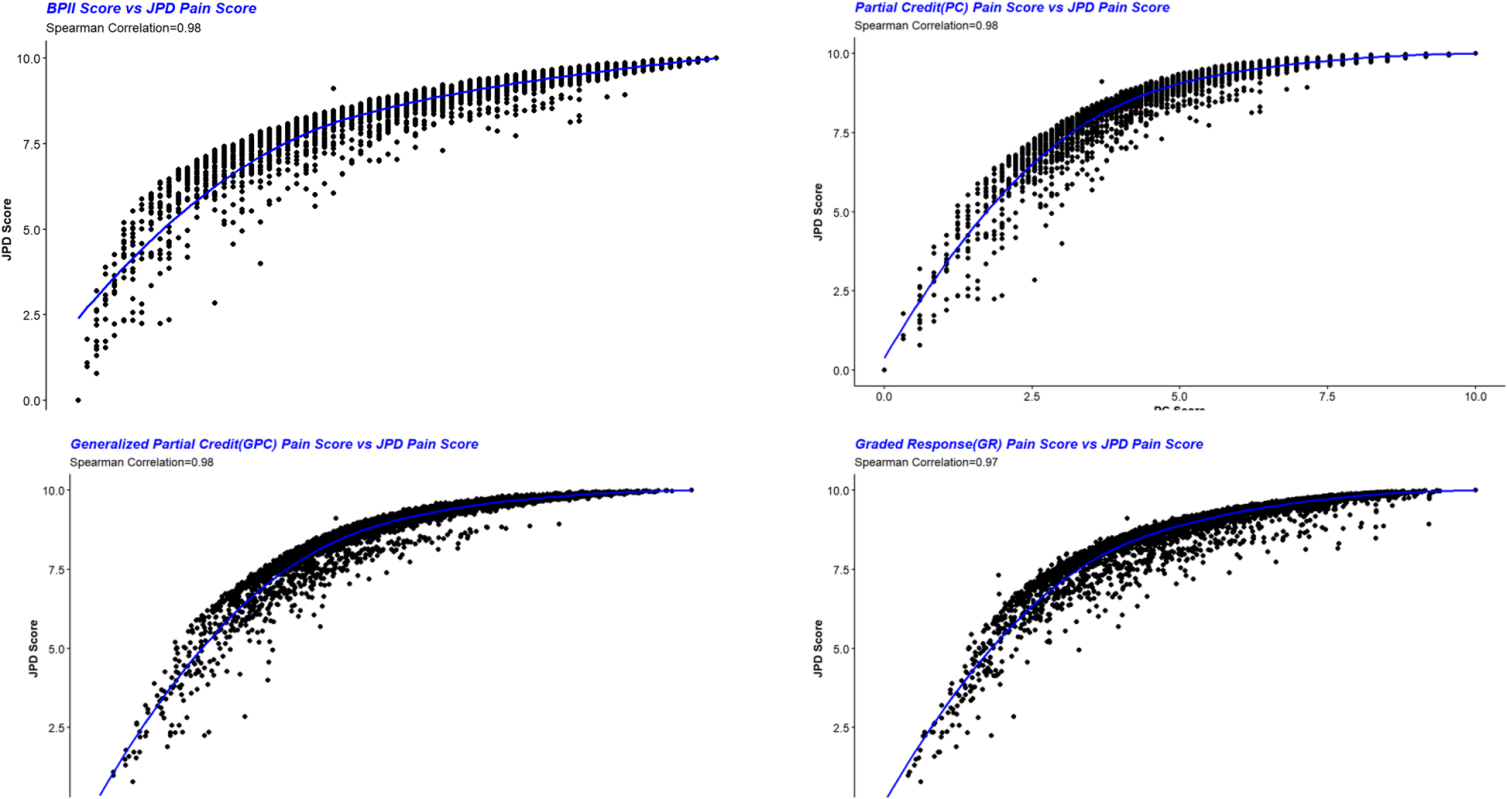
Scatterplots of JPD against the IRT models BPI-I scoring. JPD = joint probability density

We showed that using p-dimensional joint probability density function and the notion of entropy one can produce q (q < p) statistics which are most informative. This unsupervised DR theory does not require a notion of sufficiency as is common in regression DR literature. In addition, it is always possible to reduce the p-dimensional variable into a single statistic being most informative. This single statistic necessarily may not be interpretable as a latent construct or plausible summary useful to encapsulate a broader concept or characteristic. We provided the two conditions of unidirectionality and codirectionality, under which the summary measure (the index, the composite score) will rank the subjects(units) approximately the same way as the variables (the indicators) will rank. The closeness the index ranking with p rankings depends on how strong the codirectionality and unidirectionaly hold. Factor Analysis, which is based on the second moments and cross moments of the joint pdf in the case that the variance-covariances exactly or at least approximately identify the joint pdf, and when unidimensionality of the indicators, in some sense holds, the unidiemensional factor scoring and JPD will produce similar ranking. Similarly, IRT models, which are based on the joint pdf of items satisfying a set of plausible conditions, most importantly: local independence and unidimensionality, naturally should result the indices similar to the estimated JPD index. We used this result to validate our estimated index against these more established procedures. A BPI-I estimated JPD index summarizing 7 items had very good Spearman rank correlations with those produced by confirmatory factor analysis Credit and Partial credit models. the same way the variable will rank. See Fig. 7 below:

Very similar to the BPI-I indices, the MoPSI JPD estimator of 6 indicators with varying scale of measurements produced a psychiatric severity index similar to these alternative methodologies, though the strength of rank dependencies were moderate. In both cases, however, the JPD version proved more informative and granular. Conditional specification of the joint probability density has recently gained momentum in machine learning and anomaly and outlier detection, e.g., [44–47]. Results here suggest that estimating indicators’ joint probability density may also be useful for creating indices, particularly when dealing with set of multivariate data where higher moments, bigger than 2, are more informative or when IRT or classical test theory assumptions (e.g. linear dimension reduction) are not met.

BPI users should be reassured by yet another entry supporting the robustness of the BPI interference scale. While all the items appeared to be well-calibrated. Although we did not pursue it in the present paper, the JPD summary as the most informative non-linear summary might be a desirable second step in scoring when more common techniques, such as factor analysis, which is based on the linear decomposition of covariance structure, produce more than one meaningful (linear) factor. In such multidimensional (linear) latent structures, one might find lower dimensional latent construct that are non-linear. As we mentioned earlier, the single non-linear JPD index might not be interpretable as a latent trait, but when it does, it will be the best (most informative) representation of that latent construct. Were the BPI Severity and Interference subscales integrated into a single, fully informative scalar using the JPD method, investigators and clinicians could rank patients along a single pain experience continuum. This could be particularly helpful in addressing problems of rank-order when patients report low severity scores but high interference scores or vice versa.

This paper also produced a brief, novel measure of psychiatric severity based on administrative data. IRT analysis showed that emergency department visits and self-harm diagnoses best discriminated between individuals with high and low vulnerability, while the number of mental health visits varied over a much wider range of latent traits. Taken together, the six MoPSI items can discriminate individuals over both a wide and narrow range of vulnerability and have adequate measurement validity. The MoPSI’s concurrent, construct, and predictive validity have been reported elsewhere, and appear promising [34].

### Limitations

To our knowledge, using joint probability density estimation to create indices has not been previously explored. The R code, included in Additional File 1, is customizable for others’ use by substituting their variable names (and data) for ours.

Estimating multivariate densities is an old, ongoing challenge in statistics [48, 49]. Even with parametric models, higher dimensional multivariate densities have intrinsic challenges [23] and non-parametric models, even more. We used generalized linear modelling of the mean(proportion) r with simple linear combinations of indicators (no interaction, higher orders or non-linearity in the parameters) to approximate the dependencies and then estimate the node probabilities. While this choice is not necessarily a limitation, as it was confirmed by alternative scoring methods, other more elaborate multivariate density estimations can also be used [48] or even combined via a super learning recipe. Our averaging of all *p*! individual factorizations is equivalent to constructing a simple super-learner combining the estimators constructed from different methoeds.

Using Bayesian Network (BN) analysis, chain decomposition smaller than *p*! could be possible when there is specific knowledge about dependencies [50]. If lacking content knowledge, one might try to discover the conditional independencies between the indicators, then reduce the joint model to products of separate conditional probabilities. Unfortunately, BN structure discovery depends on several factors, including the optimality search criteria used, the testing methods and employed models, and search algorithms [51] and [52]. As we showed in our example in Additional File 2, Appendix Table, errors in any one of these factors can result in an unreliable model. In the present analysis, we avoided specifying the plausible dependency structures by using all possible conditional factor models, albeit at the expense of high computational cost. Fortunately, the small and large sample statistical optimality properties of JPD index, as a multivariate density estimation problem is an ongoing research agenda. However, its use under various scenarios, e.g., when a set of categorical variables (such as gender, or, race) exist, needs further studies. The use of this unsupervised DR method at the present does not, of course, addresses the variable selection questions, its applications in (time-varying) high-dimensional models, where even for high-dimensional multivariate Gaussian model surprising results are possible [53]. A particular important problem we have not addressed is the question of constructing the most informative q-dimensional linear summaries (q < *p*) where the conditional distribution of the *p*-dimensional indicators given the q linear summaries is Uniform. Such linear reductions are possible when the indicators have an elliptical distribution [54].

## Conclusions

An unsupervised marginal probabilistic dimensionality reduction method is possible. Under conditions of ordinality of the indicators, and their unidirectionality, and co-directionality, using the joint probability density one may reduce several indicators to a single scalar. The JPD method does not discard information and can handle challenging data types that may not be well-accommodated using more traditional techniques, provided an estimable multivariate density exist.

### Supplementary Information

Supplementary Material 1.

Supplementary Material 2.

## Data Availability

The data that support the findings of this study are available from the corresponding author (SN) upon reasonable request and with local Minneapolis VA Health Care System IRB permission.

## Abbreviations

AIC: Akaike information criterion
BN: Bayesian network
BIC: Bayesian information criterion
BMI: Body mass index
BPI: Short-form Brief Pain Inventory
BPI-I: Brief Pain Inventory-Interference
Dx: Diagnosis
ED: Emergency department
EPOCH: Effects of prescription opioid changes study
eRm: Extended rasch modeling R package
glm: Generalized linear model R package
GPC: Generalized partial credit model
GRD: Graded response model
ICC: Item characteristic curves
ICD-9-CM: International Classification of Diseases, Clinical Modification, 9th revision
ICD-10-CM: International Classification of Diseases, Clinical Modification, 10^t^ revision
IQR: Interquartile range
JPD: Joint probability density
MH: Mental health
MI: Mutual information
MoPSI: Manifestations of psychiatric severity index
PC: Partial credit model
Pdf: Probability density function
PIG: Poisson inverse gaussian
TRACTION: Long Term Outcomes in Veterans Requesting a Compensation Study
SAS: Statistical analysis system
SD: Standard deviation
Vul. Dist: Vulnerability distribution
