# Supplementary material for: Using joint probability density to create most informative unidimensional indices: a new method using pain and psychiatric severity as examples
Source: BMC Med Res Methodol. 2024 Aug 6;24:171. doi: 10.1186/s12874-024-02299-y (PMC11301985; doi:10.1186/s12874-024-02299-y)
Supplement: Supplementary file 1 — Supplementary Material 1. [file 12874_2024_2299_MOESM1_ESM.docx]

**Additional File 1.**

**R Codes for BPI-I and MoPSI model specification and estimation of their joint probability density**

1. **BPII Score**

dd=read.csv("R://YourData")

#Step 1: Create vectors: Let t stand for the # of variable types in your set of variables. Create a vector of all variable names and (t-1) other vectors for each type of variable, except for an arbitrary last type. Variable types could be count, continuous, and dichotomous, to name a few. For our BPII severity score example, we need only one total vector that contain all the variable names, since each BPII severity score variable is of the same type. The BPII variables will be viewed as continuous, ranging from 0 to 10. If there are 2 types of variables, as in our MoPSI example (counts and dichotomous), one will need one additional vector.

varname <-c("T0_BPII1", "T0_BPII2", "T0_BPII3", "T0_BPII4", "T0_BPII5", "T0_BPII6", "T0_BPII7")

#Step 2: Create a matrix containing all permutations (varmat) of the ordering of your variables (which variable gets predicted first given all the remaining, and so forth). Note that the # of permutations increases quickly. For our BPII severity score example with 7 indicators and 11 response options, the number of permutations is 7!=5,040 , resulting in a 5040x7 matrix . In our MoPSI example, there are 6 variables for 6!=720 permutations, resulting in a 720x6 matrix.

permutations <- function(n){

if(n==1){

return(matrix(1))

} else {

sp <- permutations(n-1)

p <- nrow(sp)

A <- matrix(nrow=n*p,ncol=n)

for(i in 1:n){

A[(i-1)*p+1:p,] <- cbind(i,sp+(sp>=i))

}

return(A)

}

}

# Note: Number of total vars=7=n, in our BPII severity score example

varmat <- matrix(varname[permutations(7)],ncol=7)

# Total number of subjects=8889, in our BPII example

sevfit=rep(NA,8889) #place holder vector

# Number of permutations=5040=7!, then add 1, so that the first column can be the StudyID of the patient

sevmat=matrix(rep(StudyID,5041),ncol=5041)

### The below 7 matrices are not needed unless you want to capture some values during the process

mod1mat=matrix(rep(StudyID,5041),ncol=5041)

mod2mat=matrix(rep(StudyID,5041),ncol=5041)

mod3mat=matrix(rep(StudyID,5041),ncol=5041)

mod4mat=matrix(rep(StudyID,5041),ncol=5041)

mod5mat=matrix(rep(StudyID,5041),ncol=5041)

mod6mat=matrix(rep(StudyID,5041),ncol=5041)

mod7mat=matrix(rep(StudyID,5041),ncol=5041)

#Step 3: Obtain predicted values from each of the conditionals and repeat this for each permutation for each subject. Therefore, for each subject in our case, there would be 35,280 sevindexi values (sevindex1 to sevindex35280, 7 specific values per permutation). This is explained more in step 4.

for (i in 1:5040) {

mod1 <- glm(get(varmat[i,1])~ get(varmat[i,2])+get(varmat[i,3])+get(varmat[i,4])+get(varmat[i,5])

+get(varmat[i,6])+get(varmat[i,7]),data =na.omit(dd10.01), family = Gamma(link = "log"))

v1=1/summary(mod1)$dispersion;

fmod1=fitted(mod1)

logden1=v1*(-get(varmat[i,1])/fmod1-log(fmod1))-(v1-1)*log(get(varmat[i,1]))

mod2 <- glm(get(varmat[i,2])~ get(varmat[i,3])+get(varmat[i,4])+get(varmat[i,5])

+get(varmat[i,6])+get(varmat[i,7]),data =na.omit(dd10.01), family = Gamma(link = "log"))

fmod2=fitted(mod2)

v2=1/summary(mod2)$dispersion

logden2=v2*(-get(varmat[i,2])/fmod2-log(fmod2))-(v2-1)*log(get(varmat[i,2]))

mod3 <- glm(get(varmat[i,3])~ get(varmat[i,4])+get(varmat[i,5])+get(varmat[i,6])+get(varmat[i,7]),

data =na.omit(dd10.01), family = Gamma(link = "log"))

fmod3=fitted(mod3)

v3=1/summary(mod3)$dispersion

logden3=v3*(-get(varmat[i,3])/fmod3-log(fmod3))-(v3-1)*log(get(varmat[i,3]))

mod4 <- glm(get(varmat[i,4])~ get(varmat[i,5])+get(varmat[i,6])+get(varmat[i,7]),

data =na.omit(dd10.01), family = Gamma(link = "log"))

fmod4=fitted(mod4)

v4=1/summary(mod4)$dispersion

logden4=v4*(-get(varmat[i,4])/fmod4-log(fmod4))-(v4-1)*log(get(varmat[i,4]))

mod5 <- glm(get(varmat[i,5])~ get(varmat[i,6])+get(varmat[i,7]),data =na.omit(dd10.01),

family = Gamma(link = "log"))

fmod5=fitted(mod5)

v5=1/summary(mod5)$dispersion

logden5=v5*(-get(varmat[i,5])/fmod3-log(fmod5))-(v5-1)*log(get(varmat[i,5]))

mod6 <- glm(get(varmat[i,6])~ get(varmat[i,7]),data =na.omit(dd10.01),family = Gamma(link = "log"))

fmod6=fitted(mod6)

v6=1/summary(mod6)$dispersion

logden6=v6*(-get(varmat[i,6])/fmod6-log(fmod6))-(v6-1)*log(get(varmat[i,6]))

mod7 <- glm(get(varmat[i,7])~ 1, data =na.omit(dd10.01),family = Gamma(link = "log"))

fmod7=fitted(mod7)

v7=1/summary(mod7)$dispersion

logden7=v7*(-get(varmat[i,7])/fmod7-log(fmod7))-(v7-1)*log(get(varmat[i,7]))

#Step 4: Take the log of the predicted value and sum the sevindexterm values for each permutation. Sevindexterms are the addends listed below from log(fmod1) to log(fmod7). Sevindexi are the values of each of these terms dependent on the permutation and range from sevindex1 to sevindex35280 for each person in our BPII severity score example.

sevfit= logden7+logden6+logden5+logden4+logden3+logden2+logden1

sevmat[,i+1]=sevfit

}

#For example, let’s say for the first person, first permutation, first sevindexterm (which is log(fmod1)), we get a value 0.0004=sevindex1. Further suppose the first person’s first permutation’s sevfit value is 0.124 (the sum of sevindex1 thru sevindex7 for that person). The second permutation would be the sum of sevindex8 through sevindex14, and so forth, until we reach the last permutation (the 5040^th^ permutation of how the 7 variables could be ordered) with the sum of sevindex35274 through sevindex35280.

# Step 5: Take the average (or mean) of the 5040 sevfit values. This is the BPII severity score per patient, on the log scale.

avgsev <- data.frame(ID=sevmat[,1], PainSevIndex=rowMeans(sevmat[,-1]))

dd2 <-cbind(dd,avgsev) #This attaches each person’s BPII severity score (avgsev) to their row of data in the dd dataframe.

1. **MoPSI Score:**

#Below is the R code for MoPSI, which is a bit more complicated because we have more than 1 type of variables. In our MoPSI example, we have 2 different types of variables: counts and dichotomous.

library(gamlss) #This R package allows us to choose the Poisson inverse Gaussian (PIG) distribution for our 3 MoPSI count variables.

#Step 1: Create vectors: Let t stand for the # of variable types in your set of variables. Create a vector of all variable names and (t-1) other vectors for each type of variable, except for an arbitrary last type. If there are 2 types of variables such as in our MoPSI example (counts and dichotomous), then you would need only one additional vector.

varname <-c("MH_clinic_count", "MH_ED_UC_count", "Hospital_count", "Suicide_harm_any", "SUD_any", "ETOH_any")

#and the one additional vector of, say the count variable names

gamlssvars <- c("MH_clinic_count", "MH_ED_UC_count", "Hospital_count")

#Step 2: Create a matrix containing all permutations (varmat) of the ordering of your variables (which variable gets predicted first given all the remaining and so forth). In our MoPSI example, with 6 variables there are 6!=720 permutations.

permutations <- function(n){

if(n==1){

return(matrix(1))

} else {

sp <- permutations(n-1)

p <- nrow(sp)

A <- matrix(nrow=n*p,ncol=n)

for(i in 1:n){

A[(i-1)*p+1:p,] <- cbind(i,sp+(sp>=i))

}

return(A)

}

}

# Note: Number of total vars=6=n, in our MoPSI example

varmat <- matrix(varname[permutations(6)],ncol=6)

# Total number of subjects=912, in our example

sevfit=rep(NA,912)

# Number of permutations=720, then add 1

sevmat=matrix(rep(StudyID,721),ncol=721)

### The below 6 matrices are not needed unless you want to capture some values during the process

mod1mat=matrix(rep(StudyID,721),ncol=721)

mod2mat=matrix(rep(StudyID,721),ncol=721)

mod3mat=matrix(rep(StudyID,721),ncol=721)

mod4mat=matrix(rep(StudyID,721),ncol=721)

mod5mat=matrix(rep(StudyID,721),ncol=721)

mod6mat=matrix(rep(StudyID,721),ncol=721)

#Step 3: Obtain predicted values from each of the conditionals and repeat this for each permutation for each subject. Therefore, for each subject in this example, there would be 4,320 sevindexi values (sevindex1 to sevindex4320, 6 specific values per permutation). This is explained more in step 4.

for (i in 1:720) {

if(varmat[i,1] %in% gamlssvars) {

mod1 <- gamlss(get(varmat[i,1])~ get(varmat[i,2])+get(varmat[i,3])+get(varmat[i,4])+

get(varmat[i,5])+get(varmat[i,6]), data =na.omit(dd), family =PIG, trace = FALSE)

} else {

mod1 <- glm(get(varmat[i,1])~ get(varmat[i,2])+get(varmat[i,3])+get(varmat[i,4])+get(varmat[i,5])

+get(varmat[i,6]),data =na.omit(dd),family=binomial)

}

fmod1=fitted(mod1)

fmod1[fmod1<0]=0.000000001

mod1mat[,i+1]=fmod1

if(varmat[i,2] %in% gamlssvars) {

mod2 <- gamlss(get(varmat[i,2])~ get(varmat[i,3])+get(varmat[i,4])+

get(varmat[i,5])+get(varmat[i,6]), data =na.omit(dd), family =PIG, trace = FALSE)

} else {

mod2 <- glm(get(varmat[i,2])~ get(varmat[i,3])+get(varmat[i,4])+get(varmat[i,5])

+get(varmat[i,6]),data =na.omit(dd),family=binomial)

}

fmod2=fitted(mod2)

fmod2[fmod2<0]=0.000000001

mod2mat[,i+1]=fmod2

if(varmat[i,3] %in% gamlssvars) {

mod3 <- gamlss(get(varmat[i,3])~ get(varmat[i,4])+

get(varmat[i,5])+get(varmat[i,6]), data =na.omit(dd), family =PIG, trace = FALSE)

} else {

mod3 <- glm(get(varmat[i,3])~ get(varmat[i,4])+get(varmat[i,5])+get(varmat[i,6]),data =na.omit(dd),family=binomial)

}

fmod3=fitted(mod3)

fmod3[fmod3<0]=0.000000001

mod3mat[,i+1]=fmod3

if(varmat[i,4] %in% gamlssvars) {

mod4 <- gamlss(get(varmat[i,4])~ get(varmat[i,5])+get(varmat[i,6]), data =na.omit(dd),

family =PIG, trace = FALSE)

} else {

mod4 <- glm(get(varmat[i,4])~ get(varmat[i,5])+get(varmat[i,6]),data =na.omit(dd),family=binomial)

}

fmod4=fitted(mod4)

fmod4[fmod4<0]=0.000000001

mod4mat[,i+1]=fmod4

if(varmat[i,5] %in% gamlssvars) {

mod5 <- gamlss(get(varmat[i,5])~ get(varmat[i,6]), data =na.omit(dd), family =PIG,

trace = FALSE)

} else {

mod5 <- glm(get(varmat[i,5])~ get(varmat[i,6]),data =na.omit(dd),family=binomial)

}

fmod5=fitted(mod5)

fmod5[fmod5<0]=0.000000001

mod5mat[,i+1]=fmod5

if(varmat[i,6] %in% gamlssvars) {

mod6 <- gamlss(get(varmat[i,6])~ 1, data =na.omit(dd), family =PIG, trace = FALSE)

} else {

mod6 <- glm(get(varmat[i,6])~ 1,data =na.omit(dd),family=binomial)

}

fmod6=fitted(mod6)

fmod6[fmod6<0]=0.000000001

mod6mat[,i+1]=fmod6

#Step 4: Take the log of the predicted value and sum the sevindexterm values for each permutation. Sevindexterms are the addends listed below from log(fmod1) to log(fmod6). Sevindexi are the values of each of these terms dependent on the permutation and range from sevindex1 to sevindex4320 for each person in our MoPSI example.

sevfit=log(fmod1)+log(fmod2)+log(fmod3)+log(fmod4)+log(fmod5)+log(fmod6)

sevmat[,i+1]=sevfit

}

#As with the BPII example above, let’s imagine for the first person, first permutation, first sevindexterm (which is log(fmod1)), we get a value of 0.0004=sevindex1. Further suppose the first person’s first permutation’s sevfit value is 0.124 (the sum of sevindex1 thru sevindex6 for that person). The second permutation would be the sum of sevindex7 through sevindex12, and so forth, until we reach the last permutation (the 720^th^ permutation of how the 6 variables could be ordered) with the sum of sevindex4315 through sevindex4320.

# Step 5: Take the average (or mean) of the 720 sevfit values. This is the severity index, MoPSI, per patient, on the log scale.

avgsev <- data.frame(ID=sevmat[,1], Means=rowMeans(sevmat[,-1]))

dd2 <-cbind(dd,avgsev) #This attaches each person’s MoPSI (avgsev) to their row of data in dd dataframe.
