# Supplementary material for: Using joint probability density to create most informative unidimensional indices: a new method using pain and psychiatric severity as examples
Source: BMC Med Res Methodol. 2024 Aug 6;24:171. doi: 10.1186/s12874-024-02299-y (PMC11301985; doi:10.1186/s12874-024-02299-y)
Supplement: Supplementary file 2 — Supplementary Material 2. [file 12874_2024_2299_MOESM2_ESM.docx]

**Additional File 2.**

**Appendix**

We propose to construct an index or composite score $S(X_{1}, X_{2},\ldots,X_{p})$ that preserves all the information in the indicators. Let $f(X_{1}, X_{2},\ldots,X_{p})$ be the joint probability density or probability mass function of the *p* dependent variables. We also refer to these variables, depending on context, as items or indicators. We assume:

**A0).** Each $X_{i}$ at least have an ordinal scale and all target a unique construct.

Increasing values of each $X_{i}$ indicate higher quantity, such as disease severity,

factory productivity, or intelligence.

**A1).** *Unidirectionality:* We can always design or code $X_{i}$’s such that: $\rho\left( X_{i} , X_{j} \right)>0$, where $\rho$ is a measure of monotone correlation or association. Spearman’s rank-correlation is one such measure of monotone dependence.

**A2).** *Co-directionality:* The index $S(X_{1}, X_{2},\ldots,X_{p})$ should be designed such that: $\rho\left( X_{i} ,S(X_{1}, X_{2},\ldots,X_{p}) \right)>0$. As above, $\rho$ measuresmonotone dependence. This assumption requires the index, which summarizes the items, to rank the subjects in the same direction as each of its items.

An index$, S(X_{1}, X_{2},\ldots,X_{p})$, is *fully informative* if, given the index, the conditional distribution of $(X_{1}, X_{2},\ldots,X_{p})$ is the maximum entropy distribution. In other words, the conditional joint distribution of the indicators over the contours defined by $S\left( X_{1}, X_{2},\ldots,X_{p} \right)=s$ is uniform. The following simple observation shows that the joint probability density, probability mass function, or a monotone function of the joint probability density or mass function provides such a summary:

Assume the sample space is finite. Let $p(\mathbf{x};\theta)$ be the multivariate probability mass function and let $S_{\theta}^{-1}(s) = \{(x_{1}, x_{2},\ldots,x_{p}) : p(x;\theta) = s\}$be the level set defined by $s$.Then for any $A \subset S_{\theta}^{-1}(s)$:

$P\left( A | x\in S_{\theta}^{-1}\left( s \right) \right)= \frac{P(A)}{P(S_{\theta}^{-1}\left( s \right))}$=$\frac{\sum_{A} s}{\sum_{S_{\theta}^{-1}(s)} s}=\frac{N_{\theta}(A)}{N_{\theta}(S_{\theta}^{-1}(s) )}$ (1)

Here, $N_{\theta}(A$) and $N_{\theta}\left( S_{\theta}^{-1}\left( s \right) \right)$ are number of elements in each set, implying: $P\left( x | S_{\theta}^{-1}\left( s \right) \right)=\frac{1}{N_{\theta}(S_{\theta}^{-1}(s) )}$, for any ${x\in S}_{\theta}^{-1}(s)$; thus, $P\left( x | S_{\theta}^{-1}\left( s \right) \right)$ is a discrete uniform distribution.

The above result holds for all statistical models with more general sample spaces, provided densities can be defined with respect to a common dominating measure. We will explore these implications with some examples. For ease of exposition and for its wide applicability, we restrict our exposition to finite sample spaces.

For elliptical densities with center parameter $\mu$ and scale matrix Σ, the score is:

$f\left( x,\mu,\Sigma\right)=h\left( \left( x-\mu\right)^{'} \Sigma^{-1} \left( x-\mu\right) \right)=s$ (2)

which is equivalent to

$D^{2}=\left( x-\mu\right)'\Sigma^{-1}(x-\mu)$ (3)

provided *h* is a known monotone function. It is well-known, [50]*, that the conditional distribution of the elliptically distributed random variables given *D*^2^ is uniform over ellipses defined by $D^{2}=s$. Note that a probability mass function--or any density—is indeed a mapping of *p*-dimensional data into non-negative numbers.

**Example 1.** Consider the following simple model.$P\left( x=1 \right)=\frac{1}{4}$ , $P\left( x=2 \right)=\frac{1}{2}, P\left( x=3 \right)=\frac{\alpha}{4}$ , $P\left( x=4 \right)=\frac{1-\alpha}{4}.$Note that labels 1,2,3,4 may denote any multidimensional combination. In this case for $0<\alpha<1 and \alpha\neq1/2$: $S_{\alpha}^{-1}\left( 1/4 \right)=\left\{ 1 \right\}, S_{\alpha}^{-1}\left( 1/2 \right)=\left\{ 2 \right\},S_{\alpha}^{-1}\left( \alpha/4 \right)=\left\{ 3 \right\},S_{\alpha}^{-1}\left( (1-\alpha)/4 \right)=\left\{ 4 \right\}$ and for $\alpha=1$: $S_{\alpha}^{-1}\left( 1/4 \right)=\left\{ 1,3 \right\}$, and for $\alpha=0$: $S_{\alpha}^{-1}\left( 1/4 \right)=\left\{ 1,4 \right\}$ and for $\alpha=1/2$: $S_{\alpha}^{-1}\left( 1/8 \right)=\left\{ 3,4 \right\}$.

A simple method for parametric models like the model in example 1, is to estimate the parameters and find “plug-in” estimators of the corresponding level sets.

As mentioned previously, for this model to be used as an *index*, the component configurations corresponding to labels 1,2,3,4 should all have ordinal properties in the same direction (A1, above), and, similarly, the density values {1/4,1/2, $\frac{\alpha}{4}$ , $\frac{1-\alpha}{4}$ } should have the same monotone direction (A2, above). A simpler, but more restrictive assumption is to assume that, if the labels have the order: 1<2<3<4, then, $P\left( x=i \right)$ is increasing in i. Obviously this monotonicity requirement does not hold for every choice of $\alpha$. However, partial monotonicity, as quantified by rank-correlation measures, might be used to indicate a (weak) unidimensional index.

**Example 2.** In BPI-I and MoPSi examples we are comparing the Generalized Partial Credit model [24] (GPCM) ability scores with JPD ranking. Let us revisit the model in our setup. Let $X=(X_{1},X_{2},\ldots,X_{p})$ be the list of p items’ responses. Here, with$m_{i}$ being the number of categories for item i, $\boldsymbol{X}_{i}=(\boldsymbol{X}_{i1},\boldsymbol{X}_{i2},\ldots,\boldsymbol{X}_{im_{i}})$. Each $\boldsymbol{X}_{ij}=1,$ if the jth category is chosen for item i, and $\boldsymbol{X}_{ij}=0,$ if the jth category is not chosen. Let for a given ability $\theta$, $P_{ij}\left( \theta\right)=P_{\theta}(\boldsymbol{X}_{ij}=1),$then,

*f*$\left( \boldsymbol{x},\theta\right)=\prod_{i=1}^{i=p} \prod_{j=1}^{j=m_{i}} {P_{ij}\left( \theta\right)}^{\boldsymbol{X}_{ij}}$.

It is assumed that $P_{ij}\left( \theta\right)=\frac{e^{\sum_{k=1}^{j} a_{I}(\theta-b_{ik})}}{\sum_{c=1}^{m_{i}} e^{\sum_{k=1}^{c} a_{I}(\theta-b_{ik})}}$ . An EM algorithm is then applied to estimate the parameters of the model. In MoPSi and BPI-I examples we used

$$logf\left( \boldsymbol{x},\theta\right)=\theta\sum_{i=1}^{p} \sum_{j=1}^{c} \boldsymbol{X}_{ij}{ja}_{I}-{\sum_{\boldsymbol{i=1}}^{\boldsymbol{p}} \sum_{\boldsymbol{j=1}}^{\boldsymbol{c}} \sum_{k=1}^{j} b_{ik}\boldsymbol{X}}_{ij}-{\sum_{\boldsymbol{i=1}}^{\boldsymbol{p}} \sum_{\boldsymbol{j=1}}^{\boldsymbol{c}} \boldsymbol{X}_{ij}log(\sum_{c=1}^{m_{i}} e^{\sum_{k=1}^{c} a_{I}\left( \theta-b_{ik} \right)}\boldsymbol{)}}=\theta\sum_{i=1}^{p} \sum_{j=1}^{c} \boldsymbol{X}_{ij}{(j-\frac{m_{i}(1+m_{i})}{2})a}_{I}-{\sum_{\boldsymbol{i=1}}^{\boldsymbol{p}} \sum_{\boldsymbol{j=1}}^{\boldsymbol{c}} \sum_{k=1}^{j} b_{ik}\boldsymbol{X}}_{ij}-\sum_{\boldsymbol{i=1}}^{\boldsymbol{p}} \sum_{\boldsymbol{j=1}}^{\boldsymbol{c}} \boldsymbol{X}_{ij}log(\sum_{c=1}^{m_{i}} e^{(-a_{I}\sum_{k=1}^{k=c} b_{ik}}))$$

Which shows that when the model holds JPD is a monotone transformation:

$$f\left( x,\theta\right)={C(\boldsymbol{x})e}^{\theta A(\boldsymbol{x})}$$

of the person’s parameter of GPCM.

**Example 2.** When (*X*_1_*,*··· *, X_n_*) is an independent and identically distributed sample of binary items then *f*$\left( \boldsymbol{x},p \right)=p^{\sum_{1}^{n} x_{i}}{(1-p)}^{n-\sum_{1}^{n} x_{i}}$, setting: $f\left( \boldsymbol{x},p \right)=c$ implies:

$\sum_{1}^{n} x_{i}=\frac{\log\left( c \right)-nlog(1-p)}{\log\left( p \right)-log(1-p)}$ (4)

and

$S_{p}^{-1}\left( c \right)=\left\{ \boldsymbol{x}: \sum_{1}^{n} x_{i}=\frac{\log\left( c \right)-nlog(1-p)}{\log\left( p \right)-log(1-p)} \right\}$ (5)

These sets are nonempty if and only if, for the given *c*, $\frac{\log\left( c \right)-nlog(1-p)}{\log\left( p \right)-log(1-p)}=j(c)$, where $j(c)$ in such a case, the uniform distribution is: $P\left( x_{1},x_{2},\ldots x_{n}|\sum_{1}^{n} x_{i}=j(c) \right)=\frac{1}{\binom{n}{j(c)}}$ over the set: $S_{p}^{-1}\left( c \right)=\left\{ \boldsymbol{x}: \sum_{1}^{n} x_{i}=j(c) \right\}.$ Note that in general *p*, the unknown population proportion, should be estimated, and the estimated score would then be a monotone function of: $\hat{p}^{\sum_{1}^{n} x_{i}}{(1-\hat{p})}^{n-\sum_{1}^{n} x_{i}}$ . However, in the above case, under the assumptions of independence and identical distribution of observations, the unidimensional sufficient statistic (that is, the total number of 1’s) is the most informative index, and subjects’ ordering remains the same, regardless of parameter values. Note that the most informative index generally is not a statistic—that is, it is not a parameter free, data-dependent function. Hence, based on the available data, the index should be estimated.

**Example 3.** Assume the multivariate observations have a joint probability density in the exponential family:$p(\underline{x},\underline{\theta})=h\left( \underline{x} \right)exp(\underline{\theta}'\underline{T}(\underline{x})$). Here,$\underline{T}\left( \underline{x} \right)=(T_{1}\left( \underline{x} \right),\ldots T_{k}\left( \underline{x} \right))$ is the joint sufficient statistic, while $S\left( x_{1}, x_{2},\ldots,x_{p},\theta\right)=logh\left( \underline{x} \right)+\underline{\theta}'\underline{T}(\underline{x})$ is a version of joint probability density summary score.

Multinomial distribution, as a member of this family, needs special attention, since it can be used to approximate unknown, continuous distributions. For multinomial distributions, estimating the class probabilities (*p*_1_*, p*_2_*, ... , p_k_*) is needed to find the estimated score for each subject with any given *x_i_* configuration. However, with several indicators (variables), each with a finite number of alternatives, the joint multinomial distribution will result in a high-dimensional contingency table with formidable estimation problems. For example, with ten indicators, each with only two levels, the number of parameters in the corresponding multinomial is 1023 (=2^10^ − 1).

**Relation with IRT model.** A common remedy to reduce the number of multinomial parameters is to assume that, given the parameters, indicators are locally independent.

Let us assume we have $p$ variables and $n$ subjects. All the variables (indicators) are binary. Unidirectionality of the indicators is ensured through the coding such that, for all $X_{ij}$s, $X_{ij}=1$ indicate a severer or higher situation than when $X_{ij}=0$.

Let $P\left( X_{ij}=1 \right)=p_{ij}$, the probability that *i*-th individual endorses the *j*-th variable. Note that $\log\left( \frac{p_{ij}}{1-p_{ij}} \right)$ is a one-to-one transformation of $p_{ij}$. For *n* subjects whose response are independent from each other and endorse the *p* indicators independently, the likelihood function is:

$\prod_{i=1,j=1}^{i=n,j=p} {{p_{ij}}^{x_{ij}}\left( 1-p_{ij} \right)^{1-x_{ij}}}$ (6)

Letting: $l_{ij}=\log\left( p_{ij} \right)-\log\left( 1-p_{ij} \right), \alpha=\sum_{i=1,j=1}^{i=n,j=p} \log\left( 1-p_{ij} \right)$, the log likelihood is

$\sum_{i=1,j=1}^{i=n,j=p} x_{ij}l_{ij}+\alpha$ (7)

Note both $\log\left( p_{ij} \right),\log\left( 1-p_{ij} \right)$ are negative numbers, and the score for *it*h subject is:

$S\left( i \right)=\sum_{j=1}^{j=p} x_{ij}l_{ij}+$ $\sum_{j=1}^{j=p} \log\left( 1-p_{ij} \right)$ (8)

$S\left( i \right)$is a weighted sum of all endorsements, with the weights, in turn, being the log odds of each person’s probability of endorsing event *j*. Note that the statuses with smaller probability of occurrence (e.g., self-harm) have smaller odds and higher negative log odds and hence more contribution in the index than more common status indicators. Also, if a subject does not endorse any indicator (so is healthy with respect to the *available* list of indicators), $\alpha_{i}$ is the subject’s baseline severity score.

Obviously, the $l_{ij}$ are unknown and in the present form not estimable. Rasch two-parameter model adds the assumption that: $l_{ij}=v_{i}-\delta_{j}$, a term ($v_{i}$) dependent only on the subject, but common to the indicators, and a term ($\delta_{j}$) dependent on the indicator but fixed for all subjects.

To mirror accepted interpretation of these parameters in testing situations, let us call $v_{i}$, the *vulnerability* of subject *i (ability),* and $\delta_{j},$ the *severity* of *j*th indicator (*item difficulty)*. This terminology agrees with our intent: in testing situations, the probability of correctly answering a test item depends positively on the test-taker’s ability and negatively on the item’s difficulty. Under this decomposition

$S\left( i \right)=v_{i}\sum_{j=1}^{j=p} x_{ij}-\sum_{j=1}^{j=p} x_{ij}\delta_{j}+\alpha_{i}$ (9)

Thus, a person who has higher indicators’ values accumulates more vulnerability in his or her summary severity index. However, this score is adjusted by sum of the indicators’ $with$varying values. The milder the indicators, the larger the $\delta_{j}$’s, and the smaller the final $S\left( i \right)$ wille.

**Index estimation based on the joint probability density.** Our unidimensional index construction based on the joint probability density is essentially multivariate density estimation, provided the component variables all are ordinal, unidirectional, and codirectional. Note that this co-directionality of the multivariate density with respect to its variables is not a modeling assumption: it is a statement about the frequencies of different patterns of observations. For example, if higher scores of all variables indicate severer cases, then and only then, can we use their density values as a summary index to capture the general notion of severity.

Although computationally challenging, we specified *all* possible factorizations of the joint probability density using the chain rule:

$f\left( x_{1}, x_{2},\ldots,x_{p} \right)=f(x_{1}| x_{2},\ldots,x_{p})f(x_{2}|x_{3},\ldots,x_{p})$ … $f(x_{p-1}| x_{p})$ $f\left( x_{p} \right)$ (10)

Each possible factorization captures different dependency structures, and each provides an estimate of the same joint model, $f\left( x_{1}, x_{2},\ldots,x_{p} \right)$ or—equivalently— $log(f\left( x_{1}, x_{2},\ldots,x_{p} \right))$. To utilize all the information captured by various models, we used the average of the log of these estimates: $\frac{\sum_{p!} log(\hat{f}(x_{i_{1}},x_{i_{2}},\ldots x_{i_{p}}))}{p!}$ (see equation 3, main text). To estimate the standard error of the estimates, we used non-parametric bootstrapping in the two examples. However, mathematical derivation of an approximate estimate, which we did not attempted in the can be attempted. This

Since there are $p!$ ways to factor the joint distribution, this method’s main drawback is that the number of possible models drastically increases with *p*. Rather than trying to discover any conditional dependencies, which implies deleting the conditioning variables in factored models, we may address this as follows: From each of *p* factors, we can choose the best fitted model (see example, next paragraph). Given the chosen best fit, we then will move to the second term and choose the best-fitted model for the second factor. We will continue in this manner *p*-1 times.

To see this forward procedure, assume we have three variables with the joint distribution: $f\left( x_{1}, x_{2},x_{3} \right)$. We first find the maximum likelihood estimate of

$f\left( x_{1}|x_{2},x_{3} \right)$, $f\left( {x_{2}|x}_{1},x_{3} \right), f\left( x_{3}|x_{1},x_{2} \right)$and their goodness of fit measures, e.g., the AIC. If the AIC of $\hat{f}\left( {x_{2}|x}_{1},x_{3} \right)$ is smallest, we choose the further conditional factorizations that start with $\hat{f}\left( {x_{2}|x}_{1},x_{3} \right)$. Hence, the next factor in the chain rule will be $\hat{f}\left( {x_{3}|x}_{1} \right)$ or $\hat{f}\left( {x_{1}|x}_{3} \right)$. We again use a maximum likelihood estimate and fit measures to choose between these two alternative possible factors. If $\hat{f}\left( {x_{1}|x}_{3} \right)$ has the smallest AIC, this leaves us to finally estimate $\hat{f}\left( x_{3} \right).$ Thus, the final estimated joint probability density function is $\hat{f}\left( {x_{2}|x}_{1},x_{3} \right)\hat{f}\left( {x_{1}|x}_{3} \right) \hat{f}\left( x_{3} \right).$

In our two examples, BPI-I (Brief Pain Inventory-Interference) and psych severity index , since the number of variables were small, rather than forward selection, we used all possible factorial decompositions: 5040 different conditional specifications for the 7 BPI-I items, and 72 0 different specifications for the 6 indicators of the psych severity index.

$$x_{1}=Hospital count, x_{2}=\# of Mental Health clinic visits, x_{3}=SUD indicator, x_{4}=Suicidality indicator, x_{5}=Mental Health urgent care visits,$$

$$x_{6}=ETOH indicator$$

Table 1. Mental Health Severity Estimates

| Criteria | Total | Globally Best Conditional Specification (Over 720 factorizations) |
| --- | --- | --- |
| AIC | 5957.35 | $f\left( x_{6} \right)f\left( x_{5} \vert x_{6} \right)f\left( x_{4} \vert x_{6},x_{5} \right)f(x_{2}\vert x_{6},x_{5},x_{4})f(x_{3}\vert x_{2},x_{4},x_{6},x_{5})f(x_{1}\vert x_{3},x_{2},x_{4},x_{6},x_{5})$ |
| BIC | 6072.93 | $f\left( x_{6} \right)f\left( x_{5} \vert x_{6} \right)f\left( x_{4} \vert x_{6},x_{5} \right)f(x_{2}\vert x_{6},x_{5},x_{4})f(x_{3}\vert x_{2},x_{4},x_{6},x_{5})f(x_{1}\vert x_{3},x_{2},x_{4},x_{6},x_{5})$ |
| Deviance | 5909.35 | $f\left( x_{6} \right)f\left( x_{5} \vert x_{6} \right)f\left( x_{4} \vert x_{6},x_{5} \right)f(x_{2}\vert x_{6},x_{5},x_{4})f(x_{3}\vert x_{2},x_{4},x_{6},x_{5})f(x_{1}\vert x_{3},x_{2},x_{4},x_{6},x_{5})$ |
| Criteria | Total | Best Forward Selected Conditional Specification |
| AIC | 6038.028 | $f\left( x_{2} \right)f\left( x_{6} \vert x_{2} \right)f\left( x_{3} \vert x_{6},x_{2} \right)f(x_{5}\vert x_{6},x_{2},x_{3})f(x_{1}\vert x_{2},x_{3},x_{6},x_{5})f(x_{4}\vert x_{3},x_{2},x_{1},x_{6},x_{5})$ |
| BIC | 6153.603 | $f\left( x_{2} \right)f\left( x_{6} \vert x_{2} \right)f\left( x_{3} \vert x_{6},x_{2} \right)f(x_{5}\vert x_{6},x_{2},x_{3})f(x_{1}\vert x_{2},x_{3},x_{6},x_{5})f(x_{4}\vert x_{3},x_{2},x_{1},x_{6},x_{5})$ |
| Deviance | 5990.028 | $f\left( x_{2} \right)f\left( x_{6} \vert x_{2} \right)f\left( x_{3} \vert x_{6},x_{2} \right)f(x_{5}\vert x_{6},x_{2},x_{3})f(x_{1}\vert x_{2},x_{3},x_{6},x_{5})f(x_{4}\vert x_{3},x_{2},x_{1},x_{6},x_{5})$ |

We notice that all three different criteria pick the same factorization as their optimal specification. For the sake of brevity, we did not include the corresponding table for the BPI-I case. Note the main difference between our specification (globally or forward selection) and Bayesian Network specification is a lack of elimination of any conditioning variable when factorization is applied. In Bayesian Network the elimination is part of structure detection. See the following section and associated table below.

**Bayesian Network analysis.** Bayesian Network (BN) is a representation of the joint distribution of all the random variables measured from the same subject via conditional factorization (Chain Rule). Variables are referred to as nodes and the relationship between variables is depicted via arcs connecting the nodes. A core task of BN is to discover some optimally fitted and most parsimonious conditional factorizations of this joint distribution by identifying independencies and/or conditional independencies between the nodes. Similar to factor analysis, BN structure discovery can either be done through some exploratory search algorithms optimizing some goodness of fit criteria (BIC, AIC,..), or, confirmatory by estimating and testing a hypothesized structure of independencies (conditional) between nodes.

Graphs are used to present the dependencies and independencies. If a line connects two nodes their corresponding variables are dependent. If no connecting line between the two nodes, then their corresponding variables are independent or conditionally independent if arrows are pointing to them. If conditionally independent, the variable at the tip of the arrow has been conditioned on the variable represented by the node at the end of an arrow. In the language of regression, the variable at the tip of the arrow is a "response" variable and the variable at the end of the arrow is a predictor variable. Acyclic-directed graphs have been used to present various conditional factorizations of the joint probability function.

In BNs, all the nodes can be a response and instead of one regression, we can have several regression models and their possible reduced submodels. How to go about choosing the right set of models needs a recipe (like backward, forward, or stepwise variable selection recipes in classical regression modeling). To search over all of the possible models, algorithms such as Hill Climbing (HC) and Growth and Shrink (GS) can be used.

While chain rule factorization requires substantial computing resources, it does allow one to bypass the potentially equally intensive work of testing, identifying, and excluding any independent conditionals, as is common in Bayesian Network (BN) analysis. In an alternative specification and estimation approach, we used 5 distinct BN methods (R package bnlearn [34]) for discovering the BPI-I’s joint probability density, including the Hill-Climbing optimizing algorithm [35] with AIC, BIC or Log likelihood criteria, and the Grow-Shrink algorithm using Pearson’s chi-squared or mutual information (Likelihood Ratio) tests at 0.05. Results are shown in the Table below.

**Appendix Table.**

Discovered Simplified Joint Probability Function of the Brief Pain Inventory-Interference (BPI-I) Items based on different Bayesian Network Discovery Methods.

| Method | Discovered Simplified Joint Probability Function |
| --- | --- |
| Hill-Climbing |  |
| AIC | $f\left( x_{2} \right)f\left( x_{5} \vert x_{2} \right)f\left( x_{7} \vert x_{2},x_{5} \right)f(x_{5}\vert x_{2},x_{7})f(x_{6}\vert x_{2},x_{7})f(x_{4}\vert x_{1})f\left( x_{3} \vert x_{4} \right)$ |
| BIC: | $f\left( x_{2} \right)f\left( x_{5} \vert x_{2} \right)f\left( x_{7} \vert x_{5} \right)f(x_{1}\vert x_{7})f(x_{6}\vert x_{7})f(x_{4}\vert x_{1})f\left( x_{3} \vert x_{4} \right)$ |
| Log-Likelihood | $f\left( x_{2} \right)f\left( x_{5} \vert x_{2} \right)f\left( x_{7} \vert x_{2},x_{5} \right)f\left( x_{6} \vert x_{2},x_{5},x_{7} \right)f\left( x_{1} \vert x_{2},x_{5},x_{6},x_{7} \right)f\left( x_{4} \vert x_{1},x_{2},x_{5},x_{6},x_{7} \right)$  $f(x_{3}\vert{x_{1},x}_{2},x_{4},x_{6},x_{7})$ |
| Grow-Shrink |  |
| χ^2^(undirected): | $f(x_{1}) f(x_{2}) f(x_{4})f(x_{6}) f(x_{7})f(x_{3}\vert x_{1},x_{2}) f(x_{5}\vert x_{2})$ |
| MI (undirected): | $f(x_{2})f(x_{3})f(x_{4})f(x_{6})f\left[ x_{7} \right]f(f(x_{5}\vert x_{2})$ |

AIC= *Akaike information criterion* BIC = Bayesian information criterion MI = Mutual information. $x_{i}$ = *i*th item in the Brief Pain Inventory-Interference scale (BPI-I)

As can be seen, each method returned a different estimand, suggesting that using a two-stage procedure (i.e., structure detection followed by estimation) to estimate the joint probability density is not robust.

*The refered references are in the main manuscript.
